# Recent progress in the patterning of perovskite films for photodetector applications

**DOI:** 10.1038/s41377-025-01958-z

**Published:** 2025-10-02

**Authors:** Chuantao Hu, Bo Li, Xiaoyue Wang, Chi Liu, Dongming Sun, Huiming Cheng

**Affiliations:** 1 School of Material Science and Engineering, University of Science and Technology of China, 72 Wenhua Road, Shenyang, 110016 China; 2 Shenyang National Laboratory for Materials Science, Institute of Metal Research, Chinese Academy of Sciences, 72 Wenhua Road, Shenyang, 110016 China; 3 Institute of Technology for Carbon Neutrality, Shenzhen Institute of Advanced Technology, Chinese Academy of Sciences, 1068 Xueyuan Avenue, Shenzhen, 518055 China

**Keywords:** Imaging and sensing, Lithography

## Abstract

Photodetectors, as the core devices for optical signal conversion, need to balance high efficiency, fast response, and low-cost fabrication. Perovskite, with its advantages of high carrier mobility and tunable band gaps, have become an ideal alternative to silicon-based materials. This paper systematically reviews the progress in the patterned fabrication techniques and device construction of perovskite photodetectors across various dimensional material systems. First, it introduces five mainstream patterned fabrication methods for perovskites: template-confined growth, inkjet printing, vapor deposition, seed-induced growth, and conventional photolithography. Then, the latest research on image sensors based on perovskite materials in different dimensions is discussed. Following this, the paper highlights two promising application directions with great development potential: flexible wearable devices and electrochemical vision systems. Finally, the challenges and potential solutions for the future development of patterned perovskite photodetectors are presented to guide the development of high-performance perovskite optoelectronic devices.

## Abstract (web summary)

This review provides a comprehensive overview of patterning techniques for perovskite thin films, highlights photodetectors based on dimensionally engineered perovskites, and discusses emerging applications and future challenges in the development of high-performance optoelectronic devices.

## Graphical abstract

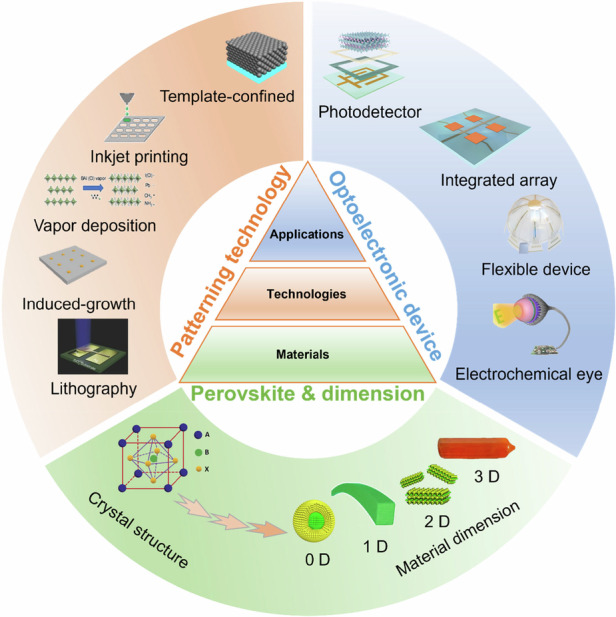

## Introduction

**Fig. 1:**
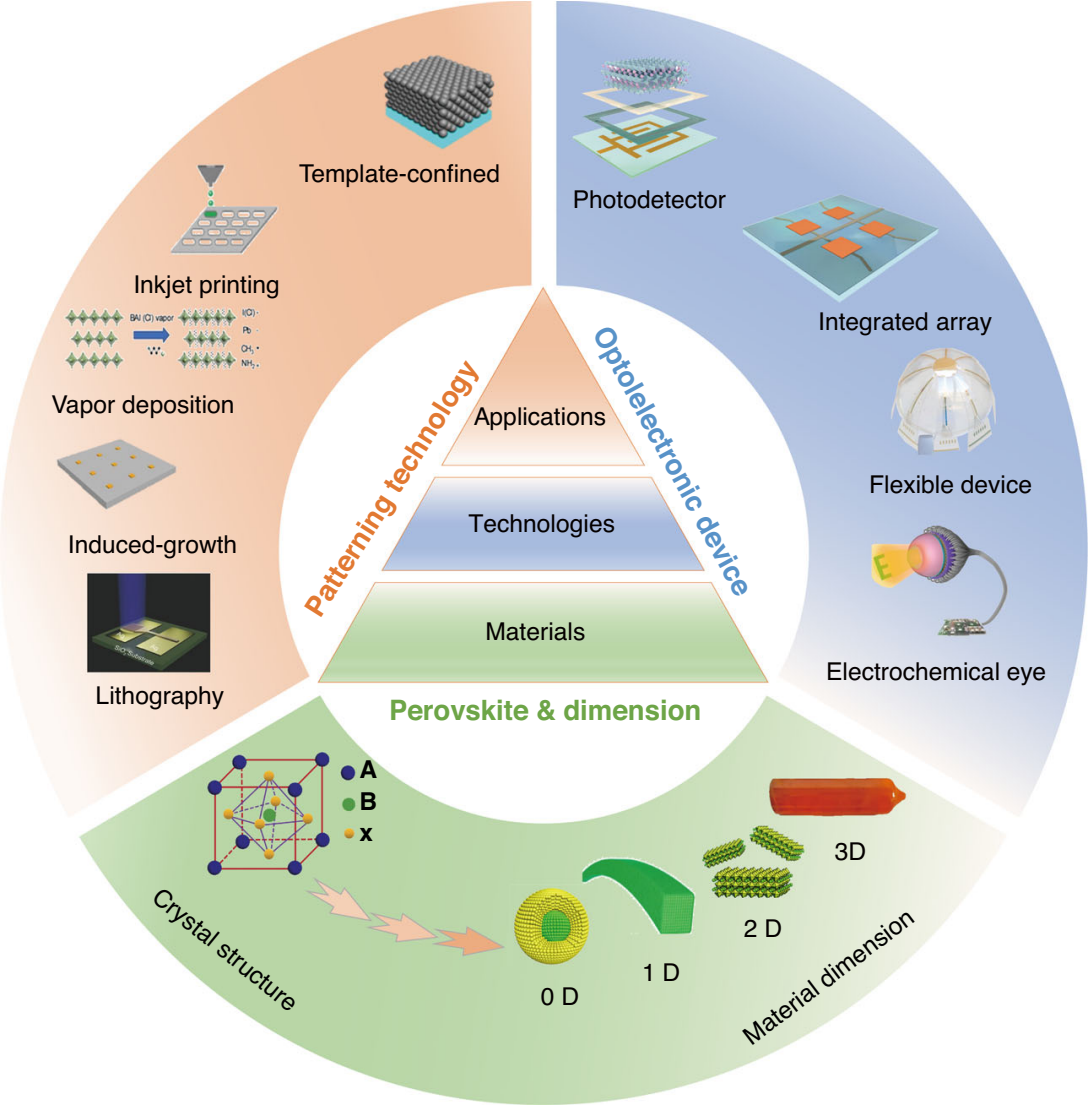
**A systematic diagram from material dimensions and patterning processes to device applications**. Perovskite structure. Reproduced with permission^15^. Copyright 2019, The Royal Society of Chemistry. Major patterning methods, including template-confined growth. Reproduced with permission^54^, Copyright 2021, Wiley-VCH GmbH, inkjet printing. Reproduced with permission^55^, Copyright 2019, WILEY-VCH Verlag GmbH & Co. KGaA. Weinheim, vapor deposition. Reproduced with permission^56^, Copyright 2019, American Chemical Society, seed-induced growth. Reproduced with permission^57^, Copyright 2018, The American Association for the Advancement of Science, as well as conventional photolithography. Reproduced with permission^58^ Copyright 2017, Optica Publishing Group. The dimensions of perovskite materials include zero-dimensional. Reproduced with permission^59^, Copyright 2019, American Chemical Society, one-dimensional. Reproduced with permission^60^, Copyright 2020, Tsinghua University Press and Springer-Verlag GmbH Germany, part of Springer Nature, two-dimensional. Reproduced with permission^61^, Copyright 2018, Royal Society of Chemistry and three-dimensional. Reproduced with permission^62^, Copyright 2023, The Authors. Small Science published by Wiley-VCH GmbH. Optoelectronic devices involve photodetector. Reproduced with permission^64^, Copyright 2023, Wiley‐VCH GmbH, integrated array. Reproduced with permission^63^, Copyright 2024, The Authors. Advanced Science published by Wiley-VCH GmbH, flexible wearable photodetectors. Reproduced with permission^64^, Copyright 2023, Wiley-VCH GmbH, as well as biomimetic electrochemical vision systems. Reproduced with permission^65^, Copyright 2020, The Author(s), under exclusive license to Springer Nature Limited

Photodetectors (PDs), which convert light signals into electrical signals^1^, are widely used in fields such as digital signal imaging^2–4^, optical communication^5,6^, and biomimetic systems^7–9^. Currently, silicon is commonly used for the construction of PDs^10^. Despite significant research advances in the use of silicon-based PDs, the materials have drawbacks such as poor light absorption^11^ and inadequate mechanical properties^12^. These drawbacks limit their ability to fully meet the rapidly evolving demands of optoelectronic devices. Perovskite is a novel material with an ABX_3_ structure (Fig. 1)^13,14^. In this structure^15^, A and X represent cations and anions, respectively. A is typically a monovalent organic cation or molecular group, while B is often a metal cation and X commonly denotes a halide anion^16,17^. A cations occupy the eight corners of an octahedron, while B cations reside at the center. X anions are located at the centers of the six faces^18^. This arrangement forms an octahedral structure around the B cation, with A cations residing within the octahedral voids. This configuration achieves charge neutrality within the crystal lattice^19^. Moreover, the structural stability of perovskite crystals is predominantly determined by the size and charge distribution of the A cation^20^. The unique crystal structure of perovskite materials gives them exceptional physicochemical properties, including a high carrier mobility^21^, high absorption coefficient^22^, tunable optical bandgap^23,24^, low defect density^25^, and long carrier diffusion lengths^26,27^. In addition, perovskite materials have a rich compositional system, and by controlling the composition of the perovskite, the material can respond to different wavelength ranges, making it suitable for constructing detectors for various wavelength ranges^28,29^. These outstanding properties make them highly promising for the fabrication and investigation of the performance of optoelectronic detectors^30–33^.

Building on the advancements in the application of perovskite materials in optoelectronic devices, patterning technology, as a key means of optimizing detector performance, is increasingly playing a significant role in the detector field. At this stage, achieving optical management through device structural design becomes crucial for further performance improvement^34^. Patterning technology is a novel approach that optimizes the performance of optoelectronic devices by constructing ordered device structures through microstructural design of the materials^35^. By using patterning processes to precisely control the morphology and microstructure of perovskite films, researchers can achieve localized light field enhancement and improved charge separation efficiency^36–38^, thereby enhancing the performance and stability of optoelectronic devices^39,40^. This technology has been preliminarily validated in fields such as flexible solar cells and PDs, offering technological support for future integration into smart wearable devices^41–44^. In 2003, Han et al. pioneered the patterning of perovskite materials^45^, by demonstrating the successful patterning of PhE-PbI_4_ thin films and achieving strong green emission at 525 nm. Subsequently, the development of patterning techniques for perovskite materials has received significant research interest^46^. Patterning technology and microstructural design techniques have also been widely used in the research on semiconductor materials and devices^47^. In the fabrication of optoelectronic detectors, the quality of the material patterning processes significantly impacts key performance metrics such as linear dynamic range, response speed, and resolution^48^. The precision of patterning processes directly determines the quality of the photosensitive material^49^. Perovskite-based PDs are expected to advance numerous emerging research fields, including biosensing, flexible wearables, and vision imaging^50–53^.

At present, many review articles focusing on patterning techniques for perovskite materials and the construction of optoelectronic detectors. Most of the existing literature concentrates on visible light optoelectronic detectors. A comprehensive review summarizing patterning techniques for both UV-Vis-NIR light and X-ray perovskite optoelectronic detectors and their applications in the optoelectronic field remains scarce. Therefore, it is warranted to conduct a timely review summarizing recent advances in their patterning techniques that bridges the gap between fabrication technologies and practical applications. This review summarizes the progress in patterning techniques of perovskite materials and their integrated application in optoelectronic devices, combining patterning processes with device construction, aiming to provide a systematic overview and reference for this field. As shown in Fig. 1, this paper first provides an in-depth analysis of the current status, advantages, and challenges of major patterning methods, including template-confined growth^54^, inkjet printing^55^, vapor deposition^56^, seed-induced growth^57^ and conventional photolithography^58^ from the perspective of process principles. Based on this, the key performance evaluation criteria for PDs are introduced, and the physical mechanisms by which patterned processes optimize device performance by controlling grain boundary density, charge transport pathways, and interface contact characteristics are revealed. For different dimensional perovskite systems, this paper provides a detailed review of the technological breakthroughs, structural advantages, and existing bottlenecks in the device construction of zero-dimensional^59^ (0D), one-dimensional^60^ (1D), two-dimensional^61^ (2D) and three-dimensional^62^ (3D) patterned perovskite materials^63^. It further introduces the exciting application prospects demonstrated by flexible wearable PDs^64^ based on patterned perovskites and biomimetic electrochemical vision systems^65^. At the end of this review, the core challenges currently faced in the field, including material environmental stability, device uniformity, and scalable fabrication, are analyzed. Solutions are proposed, such as developing new compositional systems, optimizing patterning process flows, and integrating encapsulation techniques, to provide theoretical guidance and technical roadmap references for the development of next-generation high-performance perovskite optoelectronic devices.

## Methods for patterning perovskite films

Perovskite patterning techniques primarily include five categories: template-confined growth, inkjet printing, vapor deposition, seed-induced growth, and conventional photolithography. Each method has unique characteristics and achieves precise control over the growth and patterning of perovskite materials through different fabrication approaches to meet various application needs. The following sections will introduce the principles and application features of each patterning method, starting with template-confined growth.

### Template-confined growth patterning

Template-confined growth typically uses a pre-selected material as a substrate, facilitating the nucleation and growth of the perovskite material within specifically patterned regions on the substrate. During fabrication, the substrate can either serve as a structural scaffold for the final product or be removed from the system, leaving only the perovskite structure. This offers a high degree of process flexibility. The following sections will introduce two classic template-confined growth approaches for perovskite patterning: template separation and structured templates.

#### Template-separation assisted patterning

**Fig. 2:**
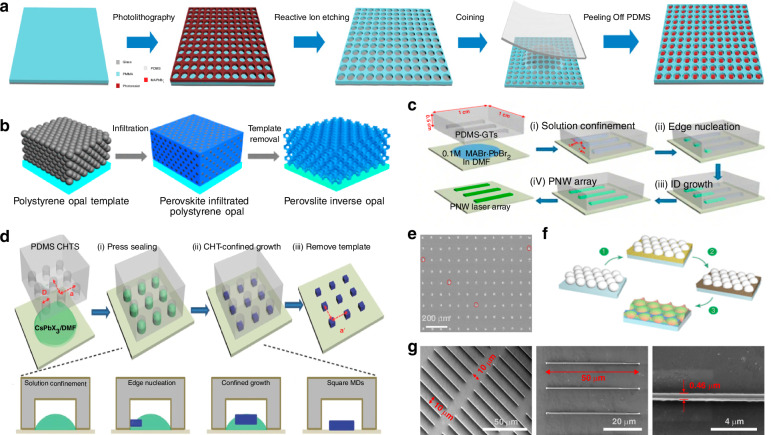
Template-separation assisted patterning. **a** Schematic of the fabrication of patterned MAPbBr_3_ single crystal arrays. Reproduced with permission^54^, Copyright 2021, Wiley-VCH GmbH. **b** Preparation protocols of the artificial template and halide perovskite photonic crystals. Reproduced with permission^67^, Copyright 2017, American Chemical Society. **c** Schematic of the preparation of MAPbBr_3_ perovskite nanowire (NW) arrays. Reproduced with permission^73^, Copyright 2017, American Chemical Society. **d** Schematic outline of the preparation of CsPbCl_3_ microdisk (MD). Reproduced with permission^74^, Copyright 2017, WILEY-VCH Verlag GmbH & Co. KGaA, Weinheim. **e** SEM image of MAPbBr_3_ single crystal arrays, the scar bar is 200 µm. Reproduced with permission^54^, Copyright 2017, American Chemical Society. **f** Diagrams of the preparation of 2D inverse opal structure perovskite photonic films. Reproduced with permission^75^, Copyright 2016, American Chemical Society. **g** Low- (left), medium- (middle), and high-magnification (right) images of MAPbBr_3_ perovskite NW arrays. Reproduced with permission^73^, Copyright 2017, American Chemical Society

Polystyrene microspheres, with its ease of molding and uniform size, has emerged as a widely used template-assist material. In 1996, Whitesides et al. pioneered the use of polydimethylsiloxane-fabricated microspheres (PDMS) for perovskite patterning, ushering its use in template-assisted patterning^66^. Luo et al. fabricated a pre-patterned polymethyl methacrylate (PMMA) micropore array template on a hydrophilic glass substrate using photolithography. Subsequently, they applied uniform pressure to hydrophobic PDMS, immersing the template completely in the perovskite precursor solution composed of methylammonium bromide (MABr) and PbBr_2_ dissolved in N, N-dimethylformamide (DMF), and pressing them onto the glass substrate. Under capillary action, the perovskite precursor solution entered the voids of the PMMA micropore array. As the solvent slowly evaporated, the perovskite nucleated at the edges of the pores and gradually grew. After keeping the structure at room temperature for 6 h, the PDMS was peeled off, yielding a fabricated rectangular methylammonium lead bromide (MAPbBr_3_) micropore plate (Fig. 2a, e)^54^. Luo et al. characterized the plate using scanning electron microscopy (SEM) and atomic force microscopy (AFM). They reported that over 96% of the plates exhibited a rectangular morphology, with individual plate thicknesses of ~500 nm and lengths ranging from 10 to 15 μm. The plates had an excellent photoresponse and good stability in an ambient atmosphere.

Plastic polystyrene is often used to make various types of molds. Tüysüz et al. used easily moldable polystyrene to fabricate polystyrene microspheres (PSMS). The authors employed centrifugation to achieve a densely packed PSMS template with monodisperse characteristics. A perovskite precursor solution, composed of 1 M methylammonium halide and 1 M lead (II) halide dissolved in dimethyl sulfoxide (DMSO), was then injected into the prepared template. Centrifugation ensured thorough contact between the microspheres and the precursor solution. Subsequent annealing followed by immersion of the resulting film in a toluene solution removed the polystyrene microspheres, yielding a patterned 3D organic-inorganic halide perovskite thin film (Fig. 2b)^67^. SEM image analysis showed that the polystyrene microspheres used in the fabrication were effectively removed, and the 3D structure formed by the polystyrene microsphere template was well-preserved, resulting in a honeycomb-like spatial arrangement. The patterned CH_3_NH_3_PbI_3_ (MAPbI_3_) perovskite 3D photonic crystals fabricated by template-confined growth exhibited excellent order and a distinct photonic band gap. The authors stated that the position of the photonic band gap could be controlled by adjusting the size of the polystyrene microspheres. Subsequently, Tüysüz et al. used the same fabrication method with larger PSMS to create a distributed feedback laser based on a 3D organic halide perovskite thin film. The resulting laser demonstrated good long-term stability under pulsed laser excitation at 1.6 mJ cm^−2^ ^68^. It is very important to study the long-term stability of perovskites. In certain patterning processes, specific protective measures can effectively prevent perovskite from decomposing under high temperature or humid conditions. However, perovskite materials still face decomposition issues in practical applications. To better understand this phenomenon, the decomposition mechanisms of perovskites and the protective mechanisms in patterning processes that mitigate decomposition will be explored. Perovskite materials are prone to degradation under environmental conditions such as light, moisture, heat, and oxygen. These factors lead to phase transitions, hydration, decomposition, and oxidation of perovskites^69^. Taking CH_3_NH_3_PbI_3_ lead halide perovskite as an example, water vapor dissolves the perovskite, and the CH_3_NH_3_^+^ cations are deprotonated by H_2_O to form CH_3_NH_3_I. CH_3_NH_3_I then decomposes into a mixture of CH_3_NH_2_ and HI. On one hand, HI can react with O_2_ to generate H_2_O and I_2_, while HI itself is unstable and easily decomposes into H_2_ and I_2_. Therefore, once CH_3_NH_3_PbI_3_ absorbs water vapor, subsequent decomposition reactions will spontaneously occur^70^. In some patterning processes, byproducts generated can form a protective barrier on the surface of the perovskite film. This barrier acts similarly to encapsulation, isolating the material from moisture and oxygen in the external environment^71^. Patterning processes can also optimize the geometry of the patterns to regulate heat conduction and stress distribution, reducing the risk of crystal fracture or phase transitions caused by local thermal expansion^72^.

In addition to fabricating 3D patterned perovskites, template-assisted patterning has been widely used to create 2D and 1D patterned perovskites. Liu et al. employed soft lithography to fabricate polydimethylsiloxane rectangular groove templates. The templates had lengths ranging from 10 to 50 μm, widths of ~1 μm, and depths of about 5 μm. The groove template was then placed on a hydrophilic substrate immersed in an DMF solution containing MAX·PbX_2_ perovskite. After applying slight pressure to the template, the perovskite solution filled the voids within the grooves. The perovskite then nucleated at the ends of the grooves and grew rapidly along their length. After allowing the solvent to completely evaporate, the template was peeled off, leaving behind 1D patterned perovskite nanowires on the hydrophilic substrate (Fig. 2c, g)^73^. Figure 2c illustrates the process developed by Liu et al. for fabricating 1D patterned perovskites using a PDMS rectangular groove template. The process involved four steps: First, an DMF solution containing MAX·PbX_2_ perovskite was confined within the groove template to form the mold. Second, perovskite nucleated at the ends of the groove. Third, the perovskite underwent 1D growth along the walls of the groove. Fourth, after the template was separated, 1D perovskite nanowires were obtained as the final product. The length and width of individual nanowires could be controlled by adjusting the dimensions of the template.

PDMS has good processability and is widely used in microfluidic systems, sensors and other fields. Fu et al. fabricated cylindrical hole templates (CHTs) made of PDMS on a silicon substrate using photolithography. After curing, the CHTs were detached from the substrate. The diameter of the PDMS-CHTs was determined by the size of silicon cylinders arranged in a square lattice on the substrate. Similar to the patterning process used by Liu et al., they first prepared an DMF solution and then immersed the PDMS-CHTs in the solution. The silicon substrate was pre-treated with octadecyltrichlorosilane to increase its hydrophobicity. They then applied slight pressure to the PDMS-CHTs to ensure that the perovskite precursor solution fully infiltrated the voids in the CHTs. After complete solvent evaporation, the fabrication of 2D patterned perovskite MD was complete (Fig. 2d)^74^. The process by which Fu et al. fabricated patterned perovskite MD, as illustrated in Fig. 2d, is as follows. First, perovskite nucleated at the edges of the PDMS-CHT substrate. Second, the formed nuclei were driven to the center of the PDMS-CHT by capillary forces. Third, perovskite grew into rectangular MD. Fourth, after separating the PDMS-CHT, 2D patterned perovskite MD were obtained as the final product. Similarly, Chen et al. employed a template-assisted patterning method to fabricate 2D patterned inverse opal structure perovskite photonic films (Fig. 2f)^75^. The authors employed a single-layer artificial opal template, introduced densely packed PSMS with diameters ranging from 100 to 2000 nm, and then added a DMSO solution to the template while spinning the substrate to remove excess solution. They heated the substrate to 100 °C to evaporate the DMSO solution while promoting the crystallization of MAPbI_3_, after which they immersed the substrate in toluene to remove the PSMS.

Template-separation assisted patterning has several advantages, including ease of template fabrication, a wide variety of possible perovskite precursors, and the ability to separate the product from the template after patterning. These advantages allow reuse of the template, significantly reducing the cost and complexity of subsequent patterning operations. However, the method also has several drawbacks for practical application. For instance, when using liquid perovskite precursors, controlling the precursor flow is difficult, leading to its non-uniform contact with the template. This disadvantage can negatively impact patterning accuracy and hinder commercial viability.

#### Structural template-assisted patterning

In the process of fabricating patterned perovskites using various template-assisted methods, some templates do not require separation from the finished perovskite and integrate with it, remaining intact on the substrate.

**Fig. 3:**
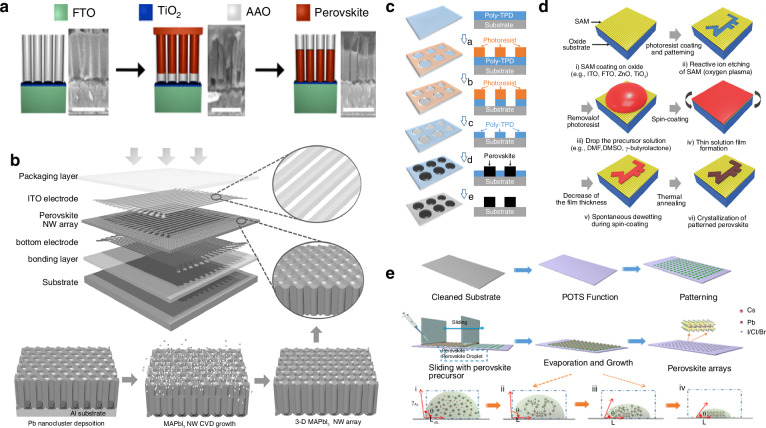
Structural template assisted patterning. **a** Synthesis of perovskite nanowire arrays in AAO: (I). MAPbI_3_ precursor solution penetrates the AAO pores, followed by spin coating and annealing (II) Sample is exposed to a DMSO/chlorobenzene solution followed by a short annealing step. Reproduced with permission^76^, Copyright 2016, American Chemical Society. **b** Layer-by-layer structure of 32 × 32 MAPbI_3_ NW image sensor with schematic of porous anodic AAO template assisted growth of a perpendicularly aligned, high-density MAPbI_3_ NW array. Reproduced with permission^77^, Copyright 2016, WILEY-VCH Verlag GmbH & Co. KGaA. Weinheim. **c** Schematics of the wetting-assisted photolithography process for a patterned MAPbI_3_ perovskite film. Reproduced with permission^78^, Copyright 2017, American Chemical Society. **d** Schematic of the spin-on-patterning (SoP) process of a inorganic-organic hybrid perovskite thin film. Reproduced with permission^79^, Copyright 2017, WILEY-VCH Verlag GmbH & Co. KGaA. Weinheim. **e** Schematic of the surface tension-controlled technique for the patterned fabrication of regular perovskite MP arrays. Reproduced with permission^80^, Copyright 2020, American Chemical Society

Mirkin et al. fabricated an anodized aluminum oxide (AAO) template with aligned cylindrical nanopores. They applied a mixed solution of DMSO and chlorobenzene to the surface of a rotating AAO template. Then, using a syringe pump to remove any residual material from the AAO surface. During this process, the perovskite precursor solution penetrated the cylindrical pores of the AAO template under the influence of capillary and centrifugal forces. They then annealed the template, during which perovskite nucleated at the bottom of the pores and subsequently grew along the pore walls, forming 1D perovskite nanowires within the cylindrical cavities of the AAO template (Fig. 3a)^76^. They claimed that this method could be used to produce an array of uniform perovskite nanowires with a dispersion of less than 10% over an area exceeding 80 cm^2^.

Similar to porous alumina, porous aluminum membranes can also serve as growth templates. Fan et al. employed a unique gas-solid-solid reaction process, utilizing a porous alumina membranes (PAM) template to fabricate 1D NW and also arranged them into a large-scale, highly ordered array (Fig. 3b)^77^. They initially prepared a PAM substrate using high-purity aluminum foil by a two-step anodization process, and then electrochemically deposited lead (Pb) at the bottom of the PAM channels using an alternating current method. Following this, they created a 2 μm thick standalone PAM embedded with Pb nanoclusters by etching away the aluminum substrate in a saturated HgCl_2_ aqueous solution. Methylammonium iodide (MAI) powder was placed at the bottom of a glass bottle, while a standalone piece of PAM/Pb was secured on a silicon substrate and positioned at the opening of the bottle. Perovskite MAPbI_3_ nanowires were obtained by the reaction the Pb and MAI vapor. Thanks to the excellent light-harvesting and anti-reflective properties of the fabricated perovskite nanowire array, they successfully constructed a high-performance image sensor with 1024 pixels.

Multi-template technology can also achieve patterning of perovskite materials. Hu et al. demonstrated a wetting-assisted photolithography (WAP) patterning process, claiming that this technique enabled the fabrication of pinhole-free hybrid perovskite thin films in arbitrary shapes^78^. As shown in Fig. 3c, they first coated a pre-cleaned glass substrate with a mixture of poly(4-butylphenyl-diphenylamine) (TPD) and chlorobenzene (CB). Using photolithography, they fabricated microstructural patterns in the photoresist layer to create the template for the WAP process, the Al_2_O_3±x_ blocking layer was fabricated on the substrate. The template was spin-drop-casted by CB to duplicate the patterning of the photoresist template on the poly-TPD layer. Then the template was dipped into NaOH aqueous solution to duplicate the patterning of the photoresist template on the Al_2_O_3±x_ layer. DMF was then spin-cast to remove the photoresist, exposing the hydrophobic surface of the patterned TPD film. Next, lead acetate (Pb(Ac)_2_) was used as the precursor, and MAI was added to DMF to spin-coat the perovskite MAPbI_3_ in the hydrophilic regions. Finally, the TPD layer was removed using the CB spin-casting method. The authors claimed that by carefully designing the photolithography template, pinhole-free hybrid perovskite thin films with arbitrary micro-patterns could be fabricated. A similar approach was used by Lee et al. who used a high-resolution SoP technique to fabricate silicon-based perovskite multiplexed image sensor arrays. They achieved perovskite thin film patterning with a resolution of ~1 µm by controlling the wetting/dewetting behavior of the perovskite precursor solution on pre-patterned hydrophilic/hydrophobic surfaces^79^. The fabrication process they described is illustrated in Fig. 3d. Initially, MAI and PbI_2_ were dissolved in DMSO to prepare the perovskite precursor solution. Bare substrates were cleaned sequentially with deionized water, isopropanol, acetone, and chloroform, followed by a 30-min ultraviolet/ozone treatment. After cleaning, the substrate was coated with a photoresist layer, that was microstructurally patterned using a photolithography system. Reactive ion etching was then used to remove the hydrophobic self-assembled monolayer selectively, exposing the oxide and rendering that region hydrophilic. The selective removal of the photoresist leads to the formation of regions with different surface energies on the patterned substrate surface, including hydrophilic oxide regions and hydrophobic self-assembled monolayer modified regions. The perovskite precursor solution was spin-coated to form a thin film whose thickness continuously decreased. Decrease of the film thickness during spin coating results in spontaneous dewetting of the solution on the hydrophobic region. Consequently, the solution on the hydrophobic interface migrates to the hydrophilic interface, and excess residual solution is removed from the substrate under the influence of centrifugal force. Finally, the template was thermally annealed to produce the 2D patterned perovskite thin film. The figure shows the steps the authors used in the fabrication of patterned perovskite thin films using the SoP process. They claim that the technique can be applied to various spin-coated mixed halide perovskite materials and deposition methods on different types of substrates. They also developed a matrix of multiplexed patterned perovskite photodiode arrays that had a high performance, and were ultra-thin, and flexible.

The hydrophilicity and hydrophobicity of the substrate surface can lead to differentiated material distribution on the substrate. Using this property, Wu et al. conceived a method for fabricating patterned perovskite films by evaporation-driven assembly, using hydrophilic/hydrophobic templates. They proposed a strategy termed surface tension-controlled assembly, which enables the low-cost, large-scale production of patterned perovskite films on pre-patterned substrates (Fig. 3e)^80^. They initially prepared the CsPbBr_3_ perovskite precursor by dissolving CsBr and PbBr_2_ in DMSO, and modified the cleaned substrates with perfluorooctyl triethoxysilane to create a hydrophobic surface before using photolithography and oxygen plasma treatment to establish periodic hydrophilic arrays on it. After spin-coating with photoresist AZ9260 and baking on a hotplate, the nonwettable substrate was covered by a designed photomask and exposed to ultraviolet (UV) light. The substrate was then immersed in acetone and ethanol to remove the photoresist, followed by plasma treatment of the non-wettable regions to create pre-patterned substrates. They then coated the patterned substrate with the perovskite precursor solution, forming an array of droplets on it, before drying, which restricted the nucleation and growth of the perovskite to the patterned hydrophilic regions. By controlling the evaporation rate of the perovskite precursor, uniformly sized 2D patterned perovskite microplate arrays were produced. They claimed that this surface tension-controlled method is applicable to substrates such as silicon wafers, glass, indium tin oxide (ITO), and SiO_2_.

In addition to using hydrophilic/hydrophobic templates for fabricating patterned perovskite films, peelable templates produced by photolithography and reactive ion etching are also commonly used for patterned perovskite fabrication. Zou et al. developed a method for producing patterned perovskite films using dry peeling enabled by poly-para-xylylene. They successfully fabricated multicolor patterns of red and green perovskite pixels on a single substrate^81^.

They first mixed CsBr, PbBr_2_, and phenethylammonium bromide (PEABr) in DMSO to prepare a green perovskite precursor. Poly-para-xylylene-C was then deposited on the substrate by chemical vapor deposition (CVD) to form a thin film. A two-step process was employed to spin-coat a negative photoresist NR9-3000 onto the poly-para-xylylene film, followed by baking. The poly-para-xylylene film was then etched using reactive ion etching and finally was mechanically peeled off using fine-tipped tweezers to obtain a 2D patterned perovskite film. They claimed that the peelable template process could achieve a resolution of around 4 μm for the patterned films. In addition, the poly-para-xylylene film effectively protected the perovskite, allowing for multicolor patterned perovskite films to be fabricated using multiple rounds of standard photolithography processes.

Unlike separable templates, structural templates are integrated with the patterned thin film during manufacture. In structural template-assisted patterning, the synthesis and patterning of the perovskite are performed simultaneously so that there is no need to separate them, eliminating a step that could damage the final product. The structural template-assisted patterning method offers high operational feasibility, and the patterned perovskite films produced have excellent resolution.

### Inkjet printing patterning

Inkjet printing patterning involves directly ejecting the target material onto the substrate through a nozzle to fabricate patterned perovskites^82^. This method offers advantages such as non-contact application, no need for masks, and minimal risk of substrate contamination^83^, making it widely used for the patterning of perovskites on various substrates. Currently, the main inkjet printing techniques can be categorized into two types: printing with perovskite precursor inks and printing using perovskite quantum dot (QD) solution inks.

#### Inkjet printing patterning based on perovskite precursor inks

Early inkjet printing methods used perovskite precursors as inks to achieve the patterning of perovskite films. In this approach, the precursor is ejected from the nozzle to specific target locations on the substrate, followed by processes such as annealing to obtain a patterned perovskite. The inks used in this method have a high stability and simple formulation, and are widely used for the fabrication of various high-precision patterned perovskites.

**Fig. 4:**
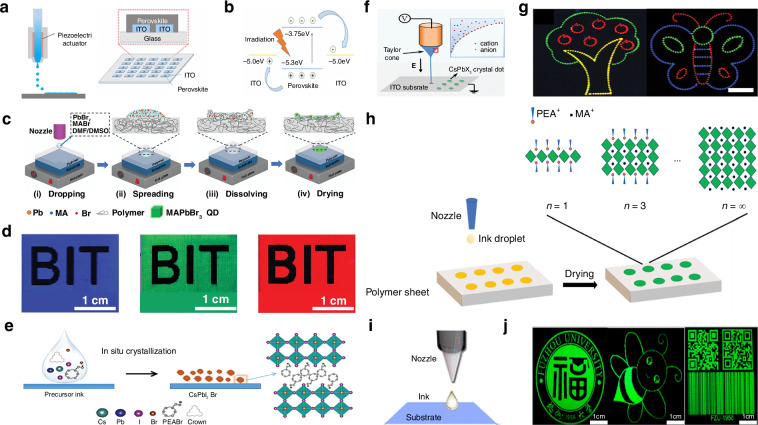
Inkjet printing patterning based on perovskite precursor inks. **a** Schematics showing the process of inkjet printing on ITO substrates and a PD array consisting of 25 pixels and the electrode–gap–electrode lateral structure of a single pixel. Reproduced with permission^84^, Copyright 2017, American Chemical Society. **b** Energy level diagram and working principle of a hybrid perovskite microwire PD. Reproduced with permission^84^, Copyright 2017, American Chemical Society. **c** Diagram of the inkjet printing strategy. Reproduced with permission^85^, Copyright 2019, WILEY-VCH Verlag GmbH & Co. KGaA. Weinheim. **d** Optical images of printed perovskite QD patterns with red, green, and blue colors. Reproduced with permission^85^, Copyright 2019, WILEY-VCH Verlag GmbH & Co. KGaA. Weinheim. **e** Schematic of perovskite transformation from CsPbX_3_ ink to a CsPbX_3_ nanocrystal. The inset is the crystal structure of mixed halide perovskites. Reproduced with permission^86^, Copyright 2019, WILEY-VCH Verlag GmbH & Co. KGaA. Weinheim. **f** Schematic of the experimental setup for the electrohydrodynamic printing system. The inset shows the enlarged Taylor cone. Reproduced with permission^86^, Copyright 2019, WILEY-VCH Verlag GmbH & Co. KGaA. Weinheim. **g** Typical photoluminescence images of the electrohydrodynamic printed microscale line arrays of an apple tree and a butterfly using three-color perovskite. Reproduced with permission^86^, Copyright 2019, WILEY-VCH Verlag GmbH & Co. KGaA. Weinheim. **h** Schematic of the preparation of quasi-2D perovskite composite sheets by inkjet printing. Reproduced with permission^87^, Copyright 2020, WILEY-VCH Verlag GmbH & Co. KGaA. Weinheim. **i** Inkjet printing of the CsPbBr_3_/polyvinylpyrrolidone composite ink. Reproduced with permission^88^, Copyright 2019, American Chemical Society. **j** Inkjet-printed, fluorescent CsPbBr_3_/polyvinylpyrrolidone nanocomposite patterns with dot-constructed microarrays. Reproduced with permission^88^, Copyright 2019, American Chemical Society

When exploring the patterning preparation method of perovskite materials, Li et al. devised a method that involved spraying droplets of perovskite precursor onto a substrate, followed by an inkjet printing technique to fabricate patterned mixed perovskite CH_3_NH_3_PbI_3_ nanowires, microwires, networks, and islands (Fig. 4a, b)^84^. They first prepared an ink by dispersing a mixed perovskite CH_3_NH_3_PbI_3_ precursor in a mixture of DMF and γ-butyrolactone. They utilized a glass substrate coated with ITO, which had been patterned in advance using photolithography after cleaning with acetone, deionized water, and ultrasonic treatment. The perovskite ink was sprayed onto the substrate using an inkjet nozzle, followed by treatment to obtain patterned mixed perovskite CH_3_NH_3_PbI_3_ products. They characterized the products using SEM, which showed that the surface of the prepared nanowires was smooth with few defects and pinholes. Compared to nanowires, the perovskite microwires had a better coverage and uniform distribution. They also noted that when the ink was ejected onto the substrate through the nozzle, most of the solvent remained in the liquid phase due to the lower temperature (about 25 °C), the precursor has enough space to flow and time to crystallize and self-assemble, favoring the large but sporadic perovskite crystallization. As the temperature increased to 45 °C, the crystallization finished before all the solvent had evaporated, causing the ink droplets to tend to form interconnected microgrid networks. When the temperature continued to rise to 75 °C, the evaporation rate surpassed the crystallization rate, holes appeared in the patterned perovskite film, resulting in the formation of island-like structures. By changing the temperature, they were able to control the morphology of the patterned perovskite. They also fabricated a micro-line PD array composed of 5 × 5 pixels using the patterned perovskite, which was then used as an imaging sensor, which achieved clear mapping of light source signals.

In addition, in order to improve the photoluminescence performance of patterned perovskite QD films, Zhong et al. proposed using inkjet printing to fabricate QD films with excellent photoluminescent properties. They designed an inkjet printing strategy that involved spraying a perovskite precursor solution onto a polymer layer to create patterned perovskite QD (Fig. 4c)^85^. They used various polymers, including PMMA, polystyrene, polyvinyl chloride (PVC), polyvinylidene fluoride, polyvinylidene chloride, cellulose acetate, and polyacrylonitrile, to fabricate polymer films on glass substrates. The polymer powders were dissolved in DMF or DMSO to prepare polymer solutions, which were then drop-cast onto the substrates to form uniform polymer layers. Different perovskite precursor solutions were used to prepare green, blue, and red patterned inks that were sprayed onto the substrates in predetermined patterns and dried by heating to obtain patterned perovskite QD films. The patterns they produced consisted of 2D arrays of patterned perovskite MD with a single-point size of ~100 µm and a thickness of about 430 nm, successfully achieving photoluminescence in green, blue, and red with a quantum yield as high as 80%. They claimed that the single-layer colored films prepared using this strategy have simpler backlight structures and lower packaging costs compared to multilayer monochromatic films. This colored film has the potential to serve as a backlight source for liquid crystal displays, while the micro-pixel array composed of perovskite QD prepared by this strategy indicates potential applications of patterned perovskite films as micro/mini-light-emitting diode light transfer films (Fig. 4d).

To improve the quality of the patterned perovskite films produced by inkjet printing, Tang et al. developed an electrohydrodynamic (EHD) inkjet printing technique that uses a simple precursor mixing technique to prepare perovskite ink, enabling the fabrication of patterned perovskite arrays by in situ crystallization without the addition of antisolvents (Fig. 4e, f)^86^. They dissolved the perovskite precursor in DMSO to obtain a precursor ink, which was then placed in a glass capillary tube, and used an ITO substrate, cleaned with acetone and deionized water, as the base. Unlike previous inkjet printing methods, they applied an EHD printing approach to achieve more precise control over the application of the precursor ink. A high voltage was applied between the both sides metal-coated capillary and the grounded substrate, causing the mobile ions in the ink to accumulate at the tip of the nozzle. When the electrostatic stress overcame the surface tension of the internal liquid interface, a fine jet was ejected from the peak of the capillary cone, then the precursor ink could crystallize on the substrate at 40 °C. By controlling the driving voltage, they could regulate the droplet size, and by moving the substrate, they could accurately position the droplets, resulting in patterned perovskite films. They optimized the driving voltage and printing distance while using a small nozzle size to fabricate a high-resolution pattern of perovskite dots with a diameter of ~5 µm. Furthermore, by altering the formulation of the perovskite precursor ink, they produced blue and red perovskite micro-wires and micro-arrays (Fig. 4g), demonstrating the potential of the EHD inkjet printing approach for full-color display applications.

Li et al. also explored the use of in-situ crystallization methods to fabricate patterned perovskites. They developed a strategy for producing 2D patterned perovskites on various polymer substrates using inkjet printing to create luminescent patterns (Fig. 4h)^87^. They selected three polymer substrates: PVC, PMMA, and polycarbonate to demonstrate the versatility of inkjet printing for fabricating patterned perovskites. The perovskite precursor ink was prepared by adding PEABr, MABr, and PbBr_2_ to DMF solvent. The precursor ink was then inkjet printed onto the substrate surface, followed by annealing of the printed patterns to evaporate the solvent and form a 2D patterned perovskite array. SEM analysis of the perovskite microplate (MP) fabricated on the three different substrates. The results showed that the spots in the PMMA samples had a distinct “coffee ring” effect, indicating that the ink tended to dissolve the PMMA. In contrast, the spots on PVC and polycarbonate were much more uniform. Stability tests on the fabricated perovskites showed that the perovskite MP on the PVC substrate were the most stable in air, moisture, and light. Additionally, due to PVC’s excellent chemical resistance, the perovskite MP on it were shown to be stable in acids, alkalies, and ethanol, making them suitable for use in various extreme conditions.

Due to the involvement of solution evaporation during inkjet printing, the evaporation rate cannot be precisely controlled, leading to the inherent “coffee ring” effect that affects the uniformity of the morphology of the printed dots after drying. Liu et al. solved this problem using a method for fabricating patterned perovskites that involved adding long-chain polyvinylpyrrolidone (PVP) to the perovskite precursor ink. This additive restricts or even eliminates outward capillary flow, thereby decreasing the “coffee ring” effect during inkjet printing (Fig. 4i)^88^. They prepared a perovskite precursor ink by adding CsBr and PbBr_2_ to a DMSO solvent, and they also incorporated a PVP additive. The precursor ink was ejected onto an ITO-coated glass substrate using a nozzle. After annealing to evaporate the solvent, they obtained a microarray of crystalline perovskite nanocomposites with dot sizes ranging from ~40 to 50 µm. They conducted a series of controlled experiments to investigate the effects of the concentration of the PVP additive and fabrication temperature on the printing of the patterned perovskites and observed that at low PVP concentrations, the slow solvent evaporation rate led to the “coffee ring” effect, while at high PVP concentrations, the high viscosity of the ink hindered printing. Consequently, they selected a moderate PVP concentration (250 mg mL^−1^) for the perovskite precursor ink and printed at different temperatures. The results indicated that at temperatures ranging from 30 to 50 °C, the instability of droplet formation resulted in discontinuous and non-uniform dot arrays. In contrast, the morphology of the perovskite array printed at 70 °C was optimal; at this temperature, the droplet flow was uniform, and the nucleation rate increased alongside the evaporation rate, significantly suppressing the “coffee ring” effect and yielding a more uniform 2D patterned perovskite morphology. Later they employed the inkjet printing of fluorescent CsPbBr_3_/PVP nanocomposites to create micron-scale dot array, resulting in a fluorescent image composed of green-emitting dot spaced 200 μm apart, which exhibited uniform and bright fluorescence at a macroscopic scale (Fig. 4j).

Clearly, utilizing perovskite precursors as ink for inkjet printing offers the advantage of operational simplicity. In such processes, inks containing perovskite QD are deposited onto substrate materials, forming target patterns after solvent evaporation. The positions of the ink deposition are easily controllable, and the ink is easy to prepare. The “coffee ring” effect that may occur during the patterning operation can be reduced by altering the ink formulation.

#### Inkjet printing patterning based on perovskite quantum dot solutions

Another method for fabricating patterned perovskites by inkjet printing involves the direct use of perovskite QD solutions as ink. When using this technique, the additives, solvents, and ink preparation significantly influence the characteristics of the product. The main technical challenge lies in how to optimize the fabrication process by adjusting these parameters to achieve uniform morphologies and high-quality patterns of the patterned perovskite.

**Fig. 5:**
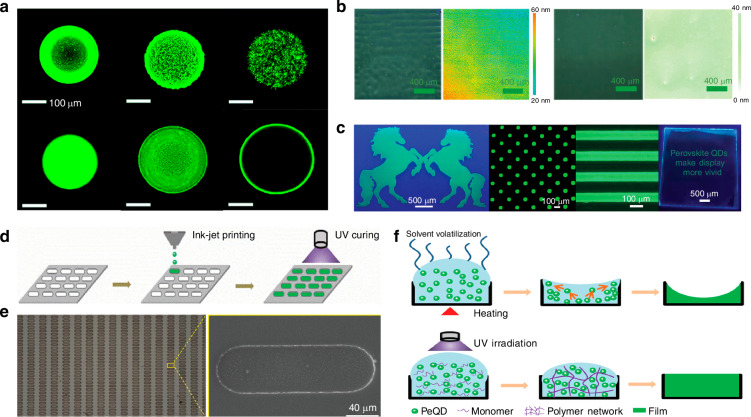
Inkjet printing patterning based on perovskite quantum dot solutions. **a** Fluorescence micrographs of inks with DOE volume contents of 20, 30, 40, 60, 70, and 80% (the scale bars are all 100 μm). Reproduced with permission^89^, Copyright 2020, Royal Society of Chemistry. **b** Optical microscope and 3D confocal microscope images of the inkjet-printed QD thin films, left: binary, right: ternary. Reproduced with permission^92^, Copyright 2022 Wiley-VCH GmbH. **c** Fluorescence optical microscope image of double horses (lighting area, 44 × 28 mm^2^), CsPbBr_3_ matrix (250 pixels per inch), QDs stripe array, and a slogan of “perovskite QDs make the display more vivid” (40 × 16 mm^2^). Reproduced with permission^92^, Copyright 2022 Wiley-VCH GmbH. **d** Schematic of the perovskite QD color conversion film fabrication process by the inkjet-printing method using UV-curing-type ink. Reproduced with permission^55^, Copyright 2019, WILEY-VCH Verlag GmbH & Co. KGaA. Weinheim. **e** SEM images of the PEROVSKITE QD film after UV curing. Reproduced with permission^55^, Copyright 2019, WILEY-VCH Verlag GmbH & Co. KGaA. Weinheim. **f** Schematic of the film forming process using thermal-curing and UV-curing inks. Reproduced with permission^55^, Copyright 2019, WILEY-VCH Verlag GmbH & Co. KGaA. Weinheim

During the solvent evaporation step when perovskite QD ink is ejected onto the substrate through a nozzle, the “coffee ring” effect can easily occur due to uneven flow of the ink droplets, resulting in a rough surface and uneven composition of the patterned perovskite product. To address this issue, Li et al. designed a strategy that incorporated high-boiling point dodecane (DOE) and low-boiling point toluene (TOL) as additives to the CsPbBr_3_ perovskite QD ink. By adjusting the ratio of these additives, they aimed to control the flow of the ink droplets, thereby eliminating the “coffee ring” effect^89^. They dispersed the perovskite CsPbBr_3_ QDs in DOE and TOL and used a nozzle to spray the ink onto an ITO-coated glass substrate to fabricate patterned perovskite microarrays and microstripes. They captured fluorescent microscopy images of inks with DOE volume contents of 20, 30, 40, 60, 70, and 80%. The results showed that when using a mixed solvent of 60 vol% DOE and 40 vol% TOL, the Marangoni flow from the low surface tension (ST) region to the high ST region counteracted and nearly canceled the natural capillary flow of the liquid. In this scenario, the internal flow of the perovskite ink droplets became more uniform, resulting in a smoother surface of the patterned perovskite array after processing. The images in Fig. 5a show fluorescent micrographs of inks with different DOE volume ratios.

During long-term storage, perovskite QD ink is prone to aggregation, and patterned perovskite is unstable in air. To address this challenge, Peng et al. proposed a perovskite QD ink formulation that includes an octane-dodecane co-solvent (in a 4:6 volume ratio) and trace amounts of oleylamine (OAm). This ink was used to fabricate a pattern of perovskite QD by inkjet printing, followed by the development of electroluminescent matrix devices^90^. They claimed that the addition of trace amounts of OAm improved the dispersion of the perovskite QD in the solvent, allowing the ink to maintain a translucent dispersion and stable storage for up to 1 month. The incorporation of the octane-dodecane co-solvent effectively suppressed the uneven flow of the ink, and thereby preventing the “coffee ring” effect. The electroluminescent matrix devices fabricated using this method achieved a pixel density of 120 pixels per inch.

In addition to these binary solvent systems designed to eliminate the influence of the “coffee ring” effect, ternary solvents have also been used. The primary distinction between them lies in the different internal flow capabilities of the ink droplets during the printing, which also leads to differences in the evaporation rates of the solvents. Compared to binary solvent systems, ternary solvent systems can better balance the boiling point and surface tension of the mixed solvent. In ternary solvent systems, long-term Marangoni flow can be generated by gradient volatilization, which helps decrease perovskite aggregation and the “coffee ring” effect^91^.

Zeng et al. reported a method for preparing cesium lead halide (CsPbX_3_) perovskite QD using ternary solvent inks, which were then used to inkjet-print patterned perovskite QD films (Fig. 5b)^92^. They constructed a ternary solvent system by introducing lower-boiling nonane into higher-boiling naphthalene and tridecane. This ternary solvent system balanced the boiling points and surface tensions of the mixed solvents, resulting in gradient evaporation while accelerating the evaporation rate of the solvent, effectively prolonging Marangoni flow and significantly suppressing the “coffee ring” effect in the printed perovskite QD films. They reported that a solution of naphthalene/tridecane/nonane (7:2:2 volume ratio) produced a uniform 2D patterned perovskite QD film without the “coffee ring” effect. They later used the ternary solvent ink system and inkjet printing to create various large-area, rigid, and flexible luminescent patterns based on different templates (Fig. 5c).

In the process of inkjet printing patterned perovskite, methods to eliminate the “coffee ring” effect include not only the addition of additives to the ink and the use of multi-solvent systems but also the UV curing of the patterned perovskite, which is a widely used approach.

Duan et al. innovatively combined inkjet printing with UV curing to fabricate 2D patterned perovskite films (Fig. 5d)^55^. They devised a method to first print perovskite QD ink onto a substrate, followed by UV irradiation of the substrate for curing, to fabricate a 2D patterned perovskite color conversion layer that was characterized by optical and scanning electron microscopies. The results indicated that the average thickness of the perovskite films was ~6 µm, with a very uniform thickness and smooth surface with no fractures or discontinuities even at the boundaries of pixel regions (Fig. 5e). The films also showed no significant “coffee ring” effect. This was attributed to the use of a UV-curable acrylate resin ink, which contains only a low concentration of solvent (Fig. 5f). During film assembly, the polymer network formed by UV curing greatly suppressed the flow within the ink droplets, which produced patterned perovskite films of uniform thickness with no “coffee ring” effect. They then used the prepared perovskite films to fabricate color conversion layers, achieving bright green color conversion from blue backlight. The patterned perovskite films produced using this technique also had excellent stability with the perovskite QD remaining well dispersed in the resin after UV curing, effectively isolating them from environmental air and moisture, so that the brightness of the films maintained over 93% of their initial brightness even after 90 days.

Unlike the inkjet printing approach that uses perovskite precursors as ink, the method employing perovskite QD as ink is faced with the susceptibility of the ink to degradation, which can occur when it is ejected onto the substrate, leading to an uneven distribution. The formulation of the ink also means that there is a higher likelihood of “coffee ring” effects occurring during inkjet printing, which can significantly impact the quality of the patterned perovskite products.

### Vapor deposition growth patterning

Traditional methods for fabricating patterned perovskite films, such as etching and photolithography, often require direct processing of the perovskite films, which can adversely affect the structural integrity of the perovskite itself and subsequently degrade its optical properties. To minimize the impact of the patterning process on the performance of the perovskite layer, vapor deposition growth has become a recent research focus. The core concept of vapor deposition for the fabrication of patterned perovskites is to control the experimental conditions so that the perovskite precursors are transformed into high-quality perovskite at predetermined target locations^93^.

**Fig. 6:**
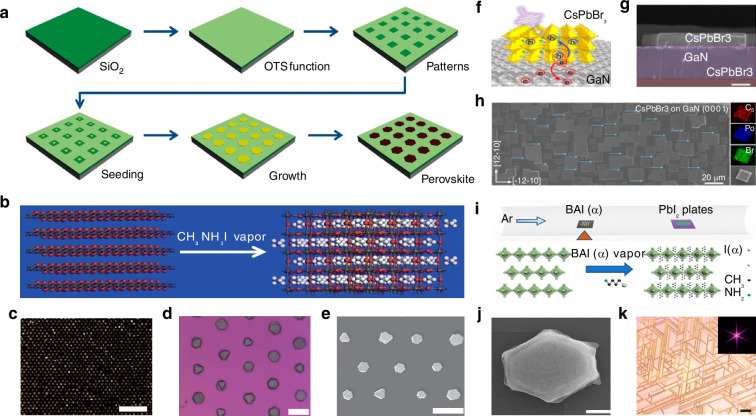
Vapor deposition growth patterning. **a** Schematic of the procedure for preparing methylammonium lead iodide perovskite plates on a patterned substrate. Reproduced with permission^46^, Copyright 2015, The American Association for the Advancement of Science. **b** Schematic of the change in lattice structure from layer PbI_2_ to tetragonal perovskite after methylammonium iodide intercalation. Reproduced with permission^46^, Copyright 2015, The American Association for the Advancement of Science. **c** Dark-field optical microscope image of perovskite MP arrays. Scale bar, 200 μm. Reproduced with permission^46^, Copyright 2015, The American Association for the Advancement of Science. **d** Higher-magnification bright-field optical microscope image of perovskite MP arrays. Scale bar, 20 μm. Reproduced with permission^46^, Copyright 2015, The American Association for the Advancement of Science. **e** SEM image of perovskite arrays. Scale bar, 20 μm. Reproduced with permission^46^, Copyright 2015, The American Association for the Advancement of Science. **f** Schematic and energy diagram of CsPbBr_3_-GaN heterojunction. Reproduced with permission^94^, Copyright 2019, Royal Society of Chemistry. **g** Cross-section SEM image of the CsPbBr_3_-GaN heterojunction. The cross-section of the CsPbBr_3_ MP shows a uniform thickness. Scale bar: 2 μm. Reproduced with permission^94^, Copyright 2019, Royal Society of Chemistry. **h** SEM image of oriented CsPbBr_3_ MP with a uniform rectangular shape epitaxial on a c-wurtzite GaN/sapphire substrate. Right: EDS images of the as-gown CsPbBr_3_ MP. Scale bar: 20 μm. Reproduced with permission^94^, Copyright 2019, Royal Society of Chemistry. **i** Setup and corresponding crystal structure changes for the gas-solid-phase intercalation process. Reproduced with permission^56^, Copyright 2019, American Chemical Society. **j** SEM images of converted (BA)_2_PbI_4_. The scale bar is 20 μm. Reproduced with permission^56^, Copyright 2019, American Chemical Society. **k** Optical microscope image of CsPbBr_3_ wire networks grown on phlogopite mica. Inset is 2D fast Fourier transform of the corresponding image, showing these wires are oriented with a hexagonal symmetry. Reproduced with permission^95^, Copyright 2017, Royal Society of Chemistry

A two-step vapor deposition process can pattern MAPbI_3_ perovskite. As shown in Fig. 6a, b, Huang et al. first immersed a silicon substrate in a octadecyltrichlorosilane (OTS) solution and then rinsed it with acetone to create a monolayer of OTS on the silicon substrate. They subsequently used photolithography and oxygen plasma treatment to selectively remove the OTS from specific locations, forming an array of microstructured hydrophilic regions. The substrate was immersed in acetone to remove the photoresist, resulting in a patterned hydrophilic/hydrophobic silicon substrate. They then deposited a dilute PbI_2_ aqueous solution onto an inclined silicon substrate and as the solution flowed over the surface, perovskite precursor droplets, formed in each hydrophilic region. After preheating the PbI_2_ nuclei on the substrate with methylammonium iodide vapor for a period, the seeded substrate was placed in a saturated PbI_2_ aqueous solution to promote further growth of the PbI_2_ nuclei. Finally, they employed methyl iodide powder as the methyl iodide source, positioned at the center of a tubular furnace, while placing the silicon substrate with the PbI_2_ nuclei array downstream. After several hours of treatment with argon as the carrier gas, MAPbI_3_ patterned perovskite arrays were formed. This gas-phase deposition method allows for the fabrication of periodically arranged perovskite arrays on wafers with a maximum diameter of 4 inches (Fig. 6c, d, e)^46^.

In addition to manufacturing 3D structured patterned perovskites, gas-phase deposition methods are also suitable for the preparation of 2D patterned perovskite film arrays. Zhang et al. proposed combining gas-phase deposition with epitaxial growth techniques to fabricate 2D patterned perovskites. They designed a method for growing highly oriented single-crystal cesium lead bromide (CsPbBr_3_) on a rectangular GaN/sapphire substrate (Fig. 6f)^94^. They employed metal-organic CVD to prepare c-plane GaN on sapphire as the substrate for perovskite growth. After cleaning the substrate with acetone, ethanol, and deionized water, they positioned it downstream in the CVD furnace while placing a mixed powder of CsBr and PbBr_2_ at the center of the furnace. Using argon as the carrier gas, they heated the system to facilitate the reaction, resulting in CsPbBr_3_ perovskite MP (Fig. 6g, h). They claimed that SEM characterization confirmed the produced CsPbBr_3_ MP exhibited a uniform rectangular shape, possessing orthorhombic or cubic phases, with dimensions ranging from a few micrometers to several tens of micrometers, and had a structural stability comparable to that of structures grown on mica substrates.

Some researchers have taken a novel approach by developing a two-step synthesis method that combines gas-phase and solution-based techniques for the preparation of 2D patterned perovskites. Li et al. reported a two-step method for producing 2D patterned perovskite MP and arrays, which integrates solution synthesis with gas-solid phase intercalation (Fig. 6i)^56^. Similar to Huang et al.^46^, they first prepared a patterned hydrophilic/hydrophobic silicon substrate using photolithography and then deposited a saturated PbI_2_ aqueous solution on it and allowed it to dry, resulting in the formation of condensed droplets of PbI_2_ in the hydrophilic regions. The silicon substrate with the grown PbI_2_ seeds was immersed in a saturated PbI_2_ aqueous solution and blow-dried with argon, to produce a silicon substrate with an array of PbI_2_ MP. Li et al. used butylammonium (BA) iodide and BA chloride powders as ammonium sources, placing them at the center of a tube furnace while positioning the silicon substrate with the grown PbI_2_ MP array downstream. Following this, the furnace was filled with argon and heated to 150 °C to complete the intercalation, resulting in the formation of (BA)_2_PbI_4-x_Cl_x_ 2D patterned perovskite films (Fig. 6j). Images indicated that the MP retained a similar hexagonal shape after the intercalation process, while its thickness significantly increased, and the surface became rougher. Characterization of the prepared (BA)_2_PbI_4-x_Cl_x_ MP using SEM, optical microscopy, and photoluminescence mapping revealed that the resulting 2D patterned perovskite array exhibited a square lattice, with individual MP distinctly hexagonal, demonstrating excellent uniformity of the final product.

One-dimensionally patterned perovskite nanowires and microwires (MWs) can also be fabricated by vapor phase epitaxy based on vapor deposition techniques. Chen et al. employed a mixture of CsX and PbX_2_ powders as the perovskite precursor that was placed in a chemical vapor deposition reactor, with freshly cleaved muscovite or phlogopite positioned in the middle to lower section of the reactor as the substrate for vapor-phase epitaxial growth^95^. By introducing argon as the carrier gas and heating the reactor to 300 °C, they achieved the synthesis of CsPbX_3_ perovskite nanowires. They noted that the freshly cleaved mica surfaces were smooth and free of dangling bonds, allowing for relaxed lattice matching conditions, which helps the growth of large, high-quality perovskite nanowires. They conducted optical microscopy characterization of the synthesized CsPbBr_3_ NW and as shown in Fig. 6k, the two samples had similar product morphologies after 2 h of CVD growth at 325 °C on p-type and m-type mica substrates. The NW were aligned parallel to the mica substrate, forming a network structure. The width of the NW was typically around 1 μm, with lengths reaching several tens of micrometers. Due to the approximately hexagonal symmetry of the mica (001) plane, the CsPbBr_3_ NWs grow in six directions with a 60° or 120° angle to each other, resulting in a uniform overall morphology.

The vapor deposition patterning method uses gaseous precursors, ensuring thorough interaction between the gas and the substrate during the process, which facilitates complete reaction. However, the process involves lattice insertion, which may induce crystal distortion and consequently degrade the quality of the patterned perovskite products.

### Seed-induced growth patterning

Seed-induced growth is a method for fabricating patterned perovskites that uses the epitaxial growth of the target product at designated sites^96^, achieving both the formation of a product array and the desired material patterning.

**Fig. 7:**
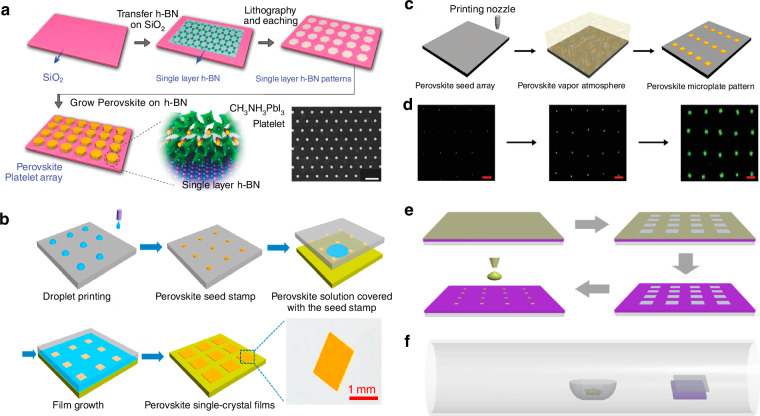
Seed-induced growth patterning. **a** Schematic of the process used to grow hexagonal lead halide perovskite (MAPbX_3_, X = Cl, Br, I) MP arrays on BN patterned films with an SEM image of the prepared MAPbX_3_ platelet array. Reproduced with permission^98^, Copyright 2016, The Authors. Published by WILEY-VCH Verlag GmbH & Co. KGaA, Weinheim. **b** Schematic of the scalable growth of perovskite single-crystal films. Reproduced with permission^57^, Copyright 2018, The American Association for the Advancement of Science. **c** Schematics of the vapor-phase fabrication of CsPbBr_3_ MP arrays. Reproduced with permission^99^, Copyright 2020, WILEY-VCH Verlag GmbH & Co. KGaA, Weinheim. **d** Optical images of the CsPbBr_3_ MP growth process. The scale bar is 50 µm. Reproduced with permission^99^, Copyright 2020, WILEY-VCH Verlag GmbH & Co. KGaA, Weinheim. **e** Illustration of seed-assisted space-confined vapor-phase growth. Reproduced with permission^100^, Copyright 2021, Wiley-VCH GmbH. **f** Schematic of the space-confined vapor-phase-growth setup. Reproduced with permission^100^, Copyright 2021, Wiley-VCH GmbH

In conventional laser patterning methods for perovskites, the direct action of the laser on the perovskite film leads to structural damage and a loss of performance while achieving the desired patterning^97^. Liu et al. innovatively used micro-patterned boron-nitride (BN) films to create a buffer layer, constructing a patterned high-quality perovskite array on a silicon substrate (Fig. 7a)^98^. They first processed the BN film using photolithography to establish growth sites, followed by the nucleation and growth of MAPbI_3_ perovskite using physical vapor deposition (PVD). Different shapes and thicknesses of patterned perovskite arrays can be fabricated through the design of the BN film patterns and by controlling the growth time. The BN film acts as an intermediary layer, providing epitaxial growth contact points between the silicon substrate and the perovskite layer, resulting in a perovskite microcrystalline array with good crystalline and high optical quality.

To address the problem of lattice mismatch during the patterning by vapor deposition, Gu et al. developed a novel synthesis method for cesium lead bromide (CsPbBr_3_) perovskite films. (Fig. 7b)^57^. They dissolved the perovskite precursor in DMSO to prepare a perovskite precursor ink, which was then printed to selected positions on the silicon substrate using inkjet printing. As the ink evaporated, ordered perovskite crystal seeds formed on the substrate. The substrate with the perovskite crystal seeds was then placed in a CVD tube furnace, where nitrogen gas was introduced, and the temperature was maintained at 650 °C for 15 min to achieve the epitaxial growth of the CsPbBr_3_. Figure 7c, d illustrates the growth process from seeds to microplate arrays. Subsequent analyses revealed that the growth sites introduced on the silicon substrate significantly lowered the crystallization barrier during perovskite growth, effectively reducing lattice mismatch and the likelihood of random nucleation. This method yielded patterned perovskites of a uniform shape and controllable size and position^99^.

The combined seed-induced growth and template-restricted growth method can be used to achieve patterning of CsPbI_3_ perovskite. Lan et al. cleaned the silicon substrate with dry nitrogen and immersed it in hexamethyldisilazane (HMDS) vapor for 30 min (Fig. 7e, f)^100^. After that, it was rinsed with acetone for 30 s to form a self-assembled HMDS hydrophobic monolayer on the substrate surface. A polymethyl methacrylate film was spin-coated on the substrate and square array pattern was fabricated by electron beam lithography. HMDS was selectively removed from specific locations to obtain periodic arrays of hydrophilic regions by oxygen plasma treatment. Then, polymethyl methacrylate was removed by ultrasonic cleaning in acetone for 10 min. CsI and PbI_2_ powders were dissolved in N, N-dimethylformamide solvent to prepare the CsPbI_3_ precursor solution. The seeded substrate was fabricated by dropping the precursor solution onto the pre-patterned silicon substrate and heating the substrate at 70 °C for 10 min to evaporate N, N-dimethylformamide solvent. A seeded silicon substrate covered by a piece of precleaned mica formed a space-confined reactor, which was placed at the center of a tube furnace. After purging by Argon gas for more than three times, the quartz tube was heated to 570 °C, and the growth time was set to be 5 min. The CsPbI_3_ microplate arrays had a smooth surface and very sharp edges, indicating their excellent crystal quality. Their research provided a new convenient and effective route to controllably grow perovskites arrays.

Seed-induced patterning can be utilized to fabricate high-quality patterned perovskites. However, the process involves many steps, including substrate preparation, inkjet printing, and epitaxial growth, which come at the cost of increased complexity and higher production expenses although achieving high-quality products.

### Conventional photolithography patterning

Photolithography has advantages such as a high processing speed, high precision, and the absence of a template, making it widely used in the fabrication of patterned perovskites. Currently, common photolithography approaches include focused ion beam (FIB) lithography, electron beam lithography (EBL), laser direct writing (LDW), laser ablation, and laser modification^101^.

#### Focused ion beam lithography patterning

**Fig. 8:**
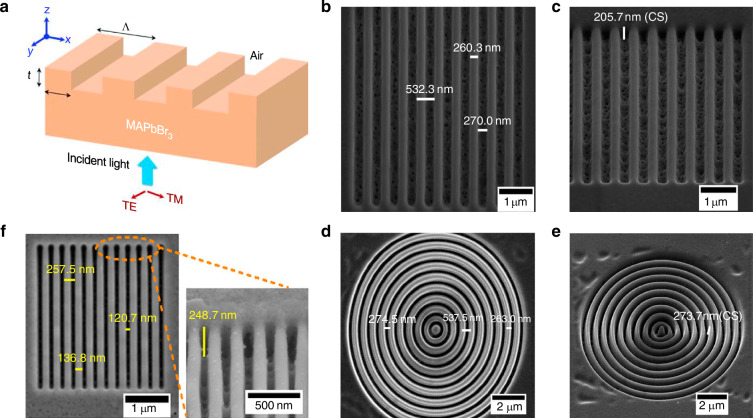
Focused ion beam lithography patterning. **a** Schematic of the MAPbBr_3_ perovskites SWG reflector. Reproduced with permission^102^, Copyright 2015, AIP Publishing. SEM images of a binary SWG **b** normal to the surface and **c** at 45°. Reproduced with permission^102^, Copyright 2015, AIP Publishing. SEM images of a circular SWG **d** normal to the surface and **e** at 45°. Reproduced with permission^102^, Copyright 2015, AIP Publishing. **f** SEM images (top-down view and 45° tilt). Reproduced with permission^103^, Copyright 2015, American Chemical Society

In the early stages, the method of using photolithography to fabricate patterned perovskites involved directly applying FIB on perovskite substrates to construct patterns by the interaction of the ion beam with the perovskite. Alias et al. reported a method for patterning methylammonium lead bromide (MAPbBr_3_) perovskite crystals using FIB technology (Fig. 8a)^102^. They applied a FIB with a Ga^+^ ion source to directly pattern the surface of MAPbBr_3_ perovskite crystals, creating binary and circular subwavelength grating (SWG) reflectors with nanometer-level precision and excellent uniformity (Fig. 8b–e). The grating thickness was ~70 nm and had a high reflectivity of about 97% at 570 nm. However, this direct ion beam process on the perovskite substrate has significant side effect: the high-energy ions used in FIB may damage the surface of perovskite crystals, and reducing the ion dose may still result in the amorphization of the crystal surface. The authors acknowledged these problems and developed a method using XeF_2_ and I_2_ gases to create an assisted environment for chemical gas-assisted focused ion beam etching in the fabrication of patterned perovskites (Fig. 8f)^103^. They fabricated SWG of equivalent specifications that had better absorption (>90%) across a broad spectral range (400–1100 nm), and improved the controllability of the manufacturing process while also improving etching precision and surface integrity.

#### Electron beam lithography patterning

To reduce damage to the perovskite substrate during the FIB patterning process, electron beam lithography (EBL) technology has been developed, in which the focused electron beam directly acts on the electron-sensitive photoresist covering the substrate under programmed control, creating the desired pattern in the photoresist layer. This pattern is then transferred to the perovskite substrate under the photoresist using fabrication processes mentioned earlier, such as the template method or chemical vapor deposition. After removing the photoresist, the required patterned perovskite is obtained.

**Fig. 9:**
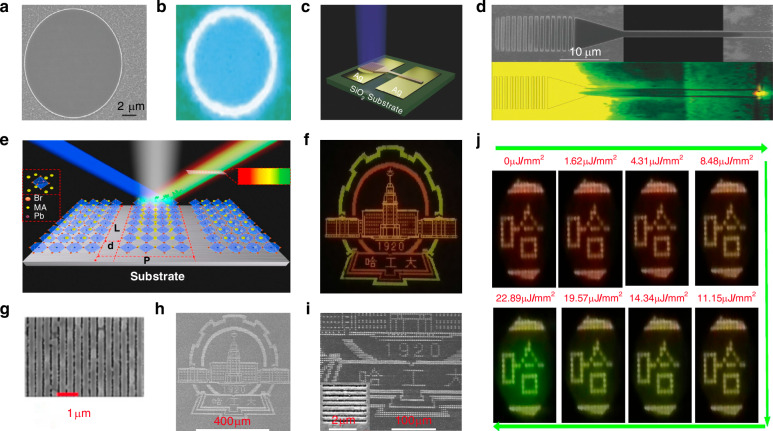
Electron beam lithography patterning. **a** Top-view SEM image of an etched perovskite MD. Reproduced with permission^58^ Copyright 2017, Optica Publishing Group. **b** Fluorescence microscopy image of a perovskite MD. Reproduced with permission^58^ Copyright 2017, Optica Publishing Group. **c** Schematic of the fabricated device. A large perovskite MP is fashioned into a grating and tapered waveguide, which is on top of two silver electrodes mounted on a glass substrate. Reproduced with permission^58^ Copyright 2017, Optica Publishing Group. **d** Top-view SEM and corresponding optical image of the device. Reproduced with permission^58^ Copyright 2017, Optica Publishing Group. **e** Schematic design of pixels and in-situ color generation by mixing extrinsic structural color and intrinsic emission color on MAPbX_3_ perovskite gratings. Reproduced with permission^104^, Copyright 2018, American Chemical Society. **f** Microscope image of the Harbin Institute of Technology logo without photon doping. Reproduced with permission^104^, Copyright 2018, American Chemical Society. **g** The top-view SEM image of the MAPbBr_3_ gratings. Reproduced with permission^104^, Copyright 2018, American Chemical Society. **h** Top-view SEM image of the Harbin Institute of Technology logo. Reproduced with permission^104^, Copyright 2018, American Chemical Society. **i** Enlarged SEM image of part of the Harbin Institute of Technology logo. The inset shows the high-resolution SEM image of one pixel of the logo. Reproduced with permission^104^, Copyright 2018, American Chemical Society. **j** Microscope images of part of the university logo at different pumping densities. Reproduced with permission^104^, Copyright 2018, American Chemical Society

Song et al. designed a top-down approach to fabricate micron and nanoscale patterned perovskite structures using EBL and inductively coupled plasma (ICP) etching on single-crystal perovskite MP (Fig. 9a, b)^58^. They employed ITO-coated glass substrates with the surface covered by a solution of MABrPbBr_2_ formed by dissolving MABr and PbBr_2_ in DMF. The pattern was then created in a PMMA resist using electron beam lithography and transferred to the organic metal halide perovskite by ICP etching. The resulting 2D patterned perovskite circular MD could be fitted to a radius of 7.34 µm and had a surface roughness of less than 100 nm (Fig. 9c, d). They claimed that the process not only enabled the fabrication of uniquely shaped patterned perovskites but also offered improved reproducibility. Qiu et al. recognized the significant potential of EBL in fabricating perovskite gratings. They reported a method that uses EBL to pattern the resist film and combined it with ICP etching to produce a 1D patterned perovskite periodic array (Fig. 9e)^104^. They fabricated color pixels with a width as small as 1.28 μm and a fixed stripe length of 3.5 μm, achieving a spatial resolution of up to 7257 dots per inch (Fig. 9f, g). They also created a sample of 530 × 610 μm^2^ to show a complex logo (Fig. 9h, i). They claimed that the 2D patterned perovskite structures produced using improved EBL could decrease the angular dependence of device photoluminescence (Fig. 9j).

#### Laser direct writing patterning

Lasers have advantages such as high energy, high brightness, and good controllability, making their use for the fabrication of patterned perovskites a recent research focus^105^. Research has shown that when a laser is applied to perovskite precursors pre-coated on the substrate, the generated heat decreases the solubility of perovskite in the precursor, leading to the crystallization of perovskite from the precursor^106^.

**Fig. 10:**
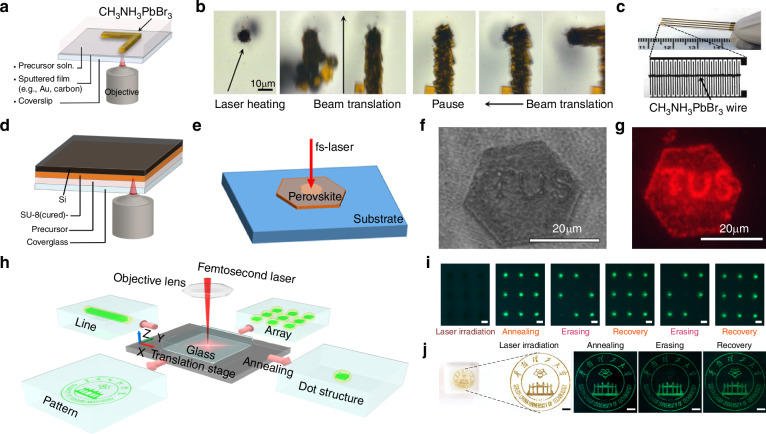
Laser direct writing patterning. **a** Schematic of the use of LDW to pattern free-form perovskite structures. Reproduced with permission^107^, Copyright 2016, American Chemical Society. **b** Time-lapse photographs showing the LDW of MAPbBr_3_ on a semitransparent carbon film. Reproduced with permission^107^, Copyright 2016, American Chemical Society. **c** A LDW MAPbBr_3_ wire drawn onto an Au interdigitated microelectrode. Reproduced with permission^107^, Copyright 2016, American Chemical Society. **d** Schematic showing the fabrication geometry for patterning on a developed and cured SU-8 photoresist microwell pattern on silicon. Reproduced with permission^107^, Copyright 2016, American Chemical Society. **e** Scheme of LDW on a perovskite nanoplate (NP)^108^. **f** Microscopic image of NP with laser patterned characters. Reproduced with permission^108^, Copyright 2019, American Chemical Society. **g** Fluorescence micrograph of patterned NP under laser excitation. Reproduced with permission^108^, Copyright 2019, American Chemical Society. **h** Schematic of the femtosecond laser writing system for sample fabrication. Reproduced with permission^109^, Copyright 2019, The Author(s), under exclusive license to Springer Nature Limited. **i** Optical images of a CsPbBr_3_ QD array during the erasure-recovery processes under UV light. Scale bars, 100 μm. Reproduced with permission^109^, Copyright 2019, The Author(s), under exclusive license to Springer Nature Limited. **j** Photograph (left) and enlarged optical microscope image (right) of a CsPbBr_3_ QD pattern. Reproduced with permission^109^, Copyright 2019, The Author(s), under exclusive license to Springer Nature Limited

Chou et al. proposed a method that uses the inverse dependence between perovskite solubility and temperature, reporting the use of lasers for localized heating of perovskite substrates to fabricate patterned MAPbBr_3_ perovskite (Fig. 10a)^107^. They first used a laser to deposit platinum in a predetermined cross-pattern on a glass substrate to act as a heating pad. A tunable continuous wave laser with a wavelength of 750 nm was then employed to irradiate the center of the platinum cross-pattern. The heat generated by the laser was conducted through the metal layer to the underlying MAPbBr_3_ perovskite, triggering its crystallization (Fig. 10b). Using this method, Chou et al. fabricated 1D patterned MAPbBr_3_ perovskite nanowires composed of interconnected crystals with an average size of 80 μm. They also used the nanowires to create a forked interdigital microelectrode array, which was used as a micro-PD (Fig. 10c, d).

Coincidentally, Wen et al. also explored the use of LDW for the fabrication of patterned perovskites. They reported a femtosecond LDW method for producing 2D patterned perovskite fluorescent NP (Fig. 10e)^108^. They employed halide-mixed formamidinium (FA) lead mixed-halide (FAPb(Br_x_I_1-x_)_3_) perovskite nanoparticles as the NP substrate, focusing an 800 nm wavelength femtosecond pulsed laser on their surface. The laser beam caused the decomposition of the perovskite NP surface (Fig. 10f, g). Using this approach, they fabricated 2D patterns of perovskite nanoparticles with various specifications.

LDW is commonly used for the fabrication of 3D patterned perovskite films. Dong et al. reported a scheme for fabricating 3D patterned perovskite films by employing femtosecond laser writing of perovskite QD inside transparent glass materials. This approach uses femtosecond laser interaction with a glass substrate to produce network dissociation and atomic rearrangement (Fig. 10h)^109^. They constructed a 3D structure of CsPbBr_3_ QD in an oxide glass matrix containing cesium, lead, and bromine using an 800 nm wavelength femtosecond laser. By changing the laser power density and exposure time, the laser’s effective range could be adjusted between 30 μm and 65 μm. They also investigated the effects of the laser on the already formed QDs, discovering that the green light emitted by the QDs could be eliminated by additional femtosecond laser irradiation, as shown in Fig. 10i, j, demonstrating the rewritability of CsPbBr_3_ QDs in a transparent medium. They also fabricated a 2D patterned perovskite array inside a 6 × 6 × 2 mm^3^ glass substrate, erasing and remanufacturing portions of the pattern, and constructed a 3D patterned CsPbBr_3_ QD perovskite film inside a 4 mm glass cube.

#### Laser-induced modification patterning

**Fig. 11:**
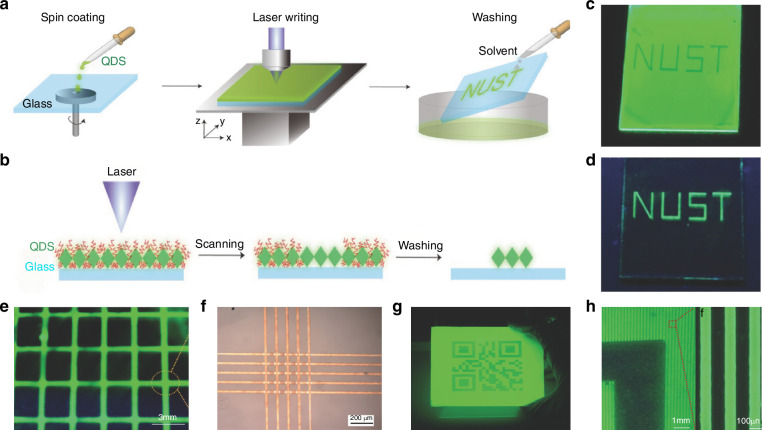
Laser-induced modification patterning. **a** Schematic of the patterning process. Reproduced with permission^110^, Copyright 2017 WILEY-VCH Verlag GmbH & Co. KGaA, Weinheim. **b** LDW mechanism for perovskite QD patterning. Reproduced with permission^110^, Copyright 2017 WILEY-VCH Verlag GmbH & Co. KGaA, Weinheim. 20 mm × 20 mm Nanjing University of Science and Technology (NUST) logo (**c**) before and (**d**) after washing under UV light. Reproduced with permission^110^, Copyright 2017 WILEY-VCH Verlag GmbH & Co. KGaA, Weinheim. **e** Macroscopic grid pattern under UV light. Reproduced with permission^110^, Copyright 2017 WILEY-VCH Verlag GmbH & Co. KGaA, Weinheim. **f** Patterns composed of a microsize cross grid. Reproduced with permission^110^, Copyright 2017 WILEY-VCH Verlag GmbH & Co. KGaA, Weinheim. **g** A patterning example on a 100 × 100 mm^2^ substrate. Reproduced with permission^110^, Copyright 2017 WILEY-VCH Verlag GmbH & Co. KGaA, Weinheim. **h** Optical image of a QD sample after washing under UV light. Reproduced with permission^110^, Copyright 2017 WILEY-VCH Verlag GmbH & Co. KGaA, Weinheim

Researchers are investigating not only the use of laser-induced perovskite crystallization in LDW but also the interaction between lasers and perovskite ligands for patterning their surface-active layers, which is a cutting-edge area of research. Zeng et al. reported a simple and rapid patterning method based on LDW that involves laser scanning to remove the surfactant surrounding perovskite QD for patterning (Fig. 11a, b)^110^. Their method can be divided into three steps: spin-coating perovskite QD, laser writing, and solvent cleaning, making it a maskless and programmable approach. They first spin-coated perovskite QD onto a glass substrate, then irradiated the perovskite QD film with a focused continuous wave laser at a wavelength of 405 nm. The laser acted on the film surface under pre-programmed control to complete the patterning process. Finally, an organic solvent was used to clean the perovskite QD substrate, leaving only the perovskite QD in the laser-irradiated areas on the substrate. By performing multiple writing operations on a single substrate, larger macroscopic patterns could be created (Fig. 11c, d). Optical microscope characterization revealed that the macroscopic pattern is composed of many microscopic lines formed by laser writing (Fig. 11e, f). They employed a laser modification scheme to create a quick response code with dimensions of 100 × 100 mm^2^, and the pattern content could be easily recognized by QR code scanning software, demonstrating the significant potential of the LDW laser modification approach for large-area display applications (Fig. 11g, h).

Conventional photolithography methods can create micron- and nanoscale patterns on the surface of perovskite materials and have developed into a relatively mature standardized system after extensive research. These lithography approaches offer significant advantages in accuracy, reliability, and commercialization. However, the interaction of lasers or electron beams with the surface of the perovskite materials inevitably causes damage, which greatly affects the quality and durability of the patterned products and further development is needed.

The above summarizes five main techniques used for patterning perovskite films and discusses their potential challenges in large-scale manufacturing. Although researchers have made significant progress in these methods in recent years, current studies mainly focus on laboratory environments or miniaturized applications. In future large-scale manufacturing processes, various patterning techniques face different challenges and bottlenecks. Template-confined growth relies on prefabricated templates to guide the material growth in specific areas, thereby achieving film patterning. However, in large-scale manufacturing, the feasibility of this method is limited by factors such as template reusability durability, template size accuracy, and its compatibility with the material. During repeated use, contamination and wear of the template may affect the uniformity of the patterns produced. In addition, templates are typically fabricated using techniques such as photolithography, and the precision and yield of the template directly impact the film quality and manufacturing efficiency. However, the high cost of manufacturing high-precision templates further limits their application in industrial production. Inkjet printing deposits material ink directly onto a substrate through a printhead to form a patterned film. In large-scale manufacturing, this method is primarily limited by printing resolution, uniformity, and ink diffusion effects. Inkjet printing struggles to achieve nanoscale resolution, and the “coffee ring” effect during droplet drying can cause uneven material distribution, affecting the final pattern quality. Moreover, the inherent characteristics of the inkjet printing process result in relatively low production efficiency, and not all materials can be formulated into stable ink solutions suitable for this technique, limiting its scalability across different material systems. Vapor deposition transforms perovskite precursors into patterned films in the target area by controlling deposition parameters. However, the main challenge of this method in large-scale fabrication lies in material compatibility, as not all materials are suitable for vapor deposition. Additionally, large-area manufacturing requires the deposition of highly uniform films; otherwise, it will significantly affect device performance. Since vapor deposition typically needs to be performed in a vacuum environment, achieving uniform deposition of large-area films and continuous manufacturing to meet high production demands remains a key challenge for this method. Seed-induced growth relies on placing seed materials at specific locations and forming the target film through epitaxial growth. In large-scale manufacturing, the precise control of seed distribution and uniformity directly determines the quality of the final patterned film. Uneven seed distribution may lead to disordered material growth, thereby reducing pattern accuracy. Additionally, this method typically requires longer growth times, resulting in lower production efficiency. Therefore, the core challenge of this technology is how to increase the growth rate while maintaining the uniformity of the film. Photolithography is currently the most mature and widely used patterning method, primarily combining photolithography and etching processes to achieve high-resolution patterned films. In large-scale manufacturing, the main bottleneck of this method lies in its dependence on high-precision photolithography equipment, masks, and etching processes. These tools are expensive, and the process steps are complex, resulting in high manufacturing costs. Additionally, some materials may be incompatible with existing photoresists or photolithography processes, requiring the development of specialized photoresists or the optimization of process parameters, which increases technical difficulty and limits the method’s applicability. Although various thin-film patterning methods face different challenges in large-scale manufacturing, the future industrialization process will require the integration of multiple techniques to overcome the limitations of any single method. For example, combining lithography with inkjet printing can enhance pattern accuracy while optimizing self-assembled templates to reduce costs. Additionally, introducing selective deposition techniques during the evaporation process can improve material utilization and minimize waste. For different application scenarios, such as flexible electronics, optoelectronic devices, and large-area integrated circuit manufacturing, it is essential to consider factors such as manufacturing cost, yield, pattern precision, and material compatibility to explore the most optimal manufacturing strategies. Integrating multiple technologies not only improves production efficiency but also accelerates the commercialization of next-generation high-performance electronic and optoelectronic devices, offering new directions for future advanced manufacturing.

## Perovskite-based photodetectors

### Introduction to photodetectors

PDs are devices that convert optical signals into electrical signals. Perovskite materials have advantages such as high absorption coefficients, tunable bandgaps, low defect densities, and long carrier diffusion lengths and lifetimes. These properties make them excellent PDs^111^, making research on them a focal point in the field of photodetection. Currently, perovskite materials used to construct PDs can be roughly divided into 0D, 1D, 2D, and 3D according to their dimensionality. PDs use a wide range of materials and structures, requiring different performance metrics for their scientific evaluation. This section provides a brief overview of these parameters and their physical significance. The evaluation metrics for ultraviolet-visible-infrared detectors differ from those for X-rays^112^. and these will be explained separately.

#### Responsivity (*R*)

For all PDs, responsivity is a critical performance parameter which characterizes the photoconversion efficiency of the PD and is defined as the ratio of the output signal to the input power, and is calculated using the following formula^113^:

$$\begin{array}{c}R=\frac{{I}_{{ph}}}{{P}_{{in}}A}=\frac{{I}_{{light}}-{I}_{{dark}}}{{P}_{{in}}A}\end{array}$$

where *I*_ph_ is the photocurrent, which is the current generated by the PD when illuminated, minus the dark current. *P*_in_ is the incident optical power density, and *A* is the effective photosensitive area of the PD.

**Fig. 12:**
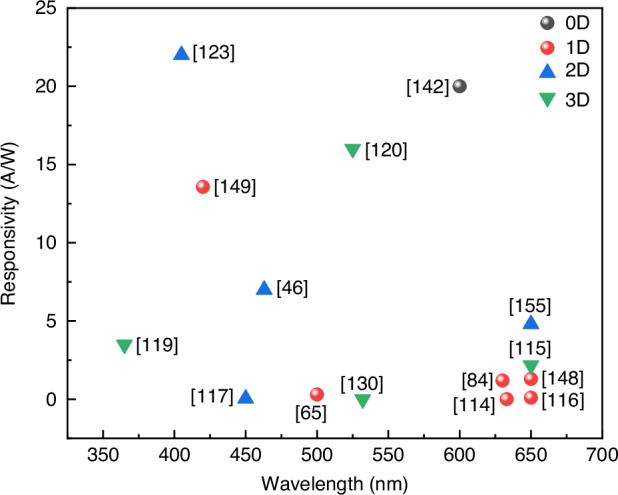
The responsivity of different PDs using patterned perovskite, the numbers in parentheses indicate the reference citations

We investigated various PDs based on patterned perovskite films, with their responsivity shown in Fig. 12. Horváth et al. reported a PD based on a 1D MAPbI_3_ perovskite nanowire cross-linked network^114^, achieving a *R* of ~5 mA W^−1^. Wang et al. fabricated a 2D patterned MAPbI_3_ perovskite array using a two-step vapor-phase method and constructed a PD with a *R* of about 7 A W^−1^ ^46^. Pan et al. developed a PD based on a 3D patterned MAPbI_3-x_Cl_x_ perovskite array, achieving a *R* of 2.17 A W^−1^ through a two-step vapor-phase method^115^.

#### Detectivity (D*)

Detectivity is a parameter that characterizes the ability of a PD to detect low-intensity light signals, typically determined by the device’s responsivity and noise. It is defined as the inverse of the noise equivalent power, which is defined as the incident optical power when the signal-to-noise ratio is equal to 1. The formula for its calculation is as follows^113^:

$${D}^{* }=\frac{{(A\varDelta f)}^{1/2}}{{NEP}}=\frac{R{(A\varDelta f)}^{1/2}}{{I}_{{noise}}}$$

where *Δf* is the bandwidth, *I*_noise_ is the noise current, *R* is the responsivity, and *A* is the effective photosensitive area of the PD.

**Fig. 13:**
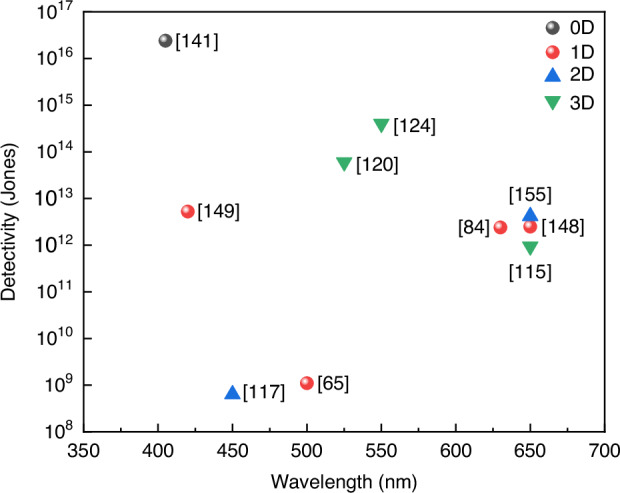
Evaluation of the detectivity performance of PDs using patterned perovskite, the numbers in parentheses indicate the reference citations

The detectivity analysis results of patterned perovskite film PDs are shown in Fig. 13. Song et al. used a template-confined growth method to construct PDs based on a patterned MAPbI_3_ perovskite micron wire network on a polyethylene terephthalate (PET) substrate, achieving a D* of 1.02 × 10^12^ Jones^116^. Li et al. used inkjet printing to fabricate a 5 × 5 pixel patterned MAPbI_3_ perovskite MW array, and the PD array had a D* of 2.39 × 10^12^ Jones^84^. Yang et al. developed a PD based on 2D patterned CsPbBr_3_ perovskite nanosheets, reaching a D* of 6.4 × 10^8^ Jones^117^.

#### External quantum efficiency (*EQE*)

The EQE is a commonly used metric for evaluating the performance of visible light PDs. It characterizes the average number of excited electrons released per incident photon when the PD is stimulated. The formula for its calculation is^118^:

$$\begin{array}{c}{EQE}=\frac{{N}_{c}}{{N}_{i}}=R\frac{{hc}}{e\lambda }\end{array}$$

where *N*_c_ is the number of photogenerated charge carriers, *N*_i_ is the number of incident photons, *R* is the resistance of the PD, *h* is Planck’s constant, *c* is the speed of light, *e* is the electronic charge, and *λ* is the wavelength of the incident light.

Research indicates that most PDs based on patterned perovskites have an exceptionally high external quantum efficiency. Xie et al. constructed a broadband PD using polycrystalline MAPbI_3_ perovskite, with an external quantum efficiency of 1.19 × 10^3^% under a bias voltage of 3 V with 365 nm light excitation^119^. Liu et al. used a liquid-phase method to develop a PD based on patterned MAPbBr_3_ single crystals, that had an external quantum efficiency of 3900% under illumination with 525 nm light and a bias voltage of 4 V^120^.

#### Photoconductive gain (*G*)

Photoconductive gain characterizes the number of charge carriers passing through the external circuit for each incident photon when the PD is operational. The formula for its calculation is^111^:

$$\begin{array}{c}G=\frac{{\tau }_{l}}{{\tau }_{t}}=\frac{{\tau }_{l}}{{d}^{2}}\mu V\end{array}$$

where *τ*_l_ is the carrier lifetime, *τ*_t_ is the transit time between the electrodes, *d* is the distance between the two electrodes, *μ* is the mobility of the majority charge carriers, and *V* is the applied voltage.

#### Response time (*τ*)

Response time characterizes the speed at which a PD responds to incident light stimuli and can be divided into rise time (*τ*_r_) and decay time (*τ*_d_). The rise time indicates the time required for the photocurrent to rise from 10% to 90% of its peak value, while the decay time refers to the time needed for it to decay from 90% to 10% of its peak value. Response time is crucial for evaluating the performance of both visible light PDs and X-ray PDs^121^.

The response times of PDs based on patterned perovskite films can reach the millisecond level. Lim et al. developed a 0D PD based on CsPbI_3_ patterned perovskite NC films, with rise and decay times of 24 ms and 29 ms, respectively^122^. Zhang et al. fabricated a PD using a two-step vapor deposition method with 2D patterned MAPbI_3_ perovskite NCs, achieving rise and decay times of less than 20 ms and 40 ms, respectively^123^.

#### Linear dynamic range (*LDR*)

The LDR characterizes the range of incident light power over which the photocurrent exhibits a linear relationship with the incident light intensity. The formula for its calculation is^111^:

$$\begin{array}{c}{LDR}=20\log \frac{{P}_{\max }}{{P}_{\min }}\end{array}$$

where *P*_max_ is the maximum incident light intensity limit of the linear range, and *P*_min_ is the minimum incident light intensity limit.

Improving the linear dynamic range of PDs is one of the key objectives for improving device performance. Yang et al. developed a novel PD based on 3D organic-inorganic hybrid patterned perovskite materials, that achieved a linear dynamic range exceeding 100 dB^124^. Zakhidov et al. used lithography to construct a PD based on 3D patterned MAPbI_3_ perovskite polycrystalline films, with a linear dynamic range of 80 dB^125^.

#### Sensitivity (*S*)

Sensitivity is an important parameter that describes the X-ray PD’s ability to detect radiation^126^. The formula for its calculation is^127^:

$$S=\frac{{I}_{{radiation}}-{I}_{{dark}}}{{DA}}$$

where *I*_radiation_ and *I*_dark_ are the output currents with and without X-ray irradiation, respectively, *D* is the X-ray dose, and *A* is the sensor area.

We surveyed the sensitivity performance of various X-ray PDs based on patterned perovskite. Shabbir et al. developed a 1D flexible X-ray PD using high-quality colloidal CsPbBr_3_ perovskite QD produced by an inkjet printing method, that achieved a sensitivity of up to 1450 µC Gy_air_^−1^ cm^−2^ under a bias voltage of 0.1 V^128^. Yu et al. constructed a 3D X-ray PD using a patterned halide double perovskite and polymer composite film as the X-ray PD, with a sensitivity of 40 μC Gy_air_^−1^ cm^−2,^^129^. Chen et al. created an X-ray PIN diode PD based on 3D patterned MAPbBr_3_ perovskite single crystals, that achieved a sensitivity of 23.6 µC Gy_air_^−1^ cm^−2,^^130^.

#### Noise

Noise is a key parameter for evaluating X-ray PDs, representing the lowest detectable signal level and being closely related to the sensitivity of the PD^131^. Noise can often induce noise current (*I*_noise_), affecting the normal operation of the device. There are four different types of noise: shot noise (*I*_shot_), thermal noise (*I*_thermal_), flicker noise (*I*_1/f_), and generation-recombination noise (*I*_g-r_). The formulas for their calculation are^121^:

$${I}_{{noise}}={({I}_{{shot}}^{2}+{I}_{{thermal}}^{2}+{I}_{1/F}^{2}+{I}_{g-r}^{2})}^{1/2}$$

$${I}_{{shot}}=\sqrt{2e{I}_{{dark}}B}$$

$${I}_{{thermal}}=\sqrt{\frac{4{kTB}}{{R}_{{sh}}}}$$

$${I}_{1/F}=I(F,B{)}_{1/F}^{2}$$

$${I}_{g-r}=I(F,B{)}_{g-r}$$

where *e* is the electron charge, *I*_dark_ is the dark current, *B* is the bandwidth, *T* is the absolute temperature, *k* is Boltzmann’s constant, *R*_sh_ is the shunt resistance of the PD, and *F* is the frequency.

#### Signal-to-noise ratio (*SNR*)

In X-ray PDs, the presence of noise current affects the accuracy of the device; thus, the SNR is used to describe the detection limit of the X-ray PD. The formula for its calculation is^132^:

$${SNR}=\frac{{J}_{s}}{{J}_{n}}$$

$${J}_{s}={J}_{{radiation}}-{J}_{{dark}}$$

$${J}_{n}=\sqrt{\frac{1}{N}\mathop{\sum }\limits_{i}^{N}{({J}_{i}-{J}_{{radiation}})}^{2}}$$

where *J*_s_ is the signal current density, *J*_n_ is the noise current density, *J*_radiation_ is the photo current density, and *J*_dark_ is the dark current density.

The performance of optoelectronic detectors depends not only on the materials used but also on the device structure. Patterning techniques can influence the materials used to construct the device as well as the shape of the materials. For example, by fabricating anti-reflective shapes, light absorption can be enhanced, increasing the device’s quantum efficiency and improving detector performance. Additionally, controlling the crystallographic orientation of the material can reduce internal defects, thereby lowering the dark current and noise of the detector, which enhances key parameters such as detectivity and responsivity. However, not every patterning technique will improve device performance. If the patterning process damages the material structure or introduces additional interface defects, it can actually degrade device performance. Specifically, we will address the following aspects:

In enhancing device quantum efficiency, surface structure optimization of the thin film material is an effective strategy. The main role of patterning techniques manifests in two aspects: first, by creating structural traps to reduce light reflection losses, thereby enhancing light capture ability and improving light utilization efficiency. Hu et al. used nanoimprint lithography to fabricate high-performance nanoscale patterned perovskite PD. The study showed that the crystallinity and optical properties of spin-coated MAPbI_3_ perovskite were significantly improved after nanoimprinting. Compared to traditional un-imprinted thin-film devices, the nanoimprinted metal-semiconductor-metal PD exhibited superior performance. Further investigation revealed that the geometric morphology of the nanopatterns had a significant impact on the optoelectronic properties. Among them, the PD based on the nanopatterned grating structure demonstrated the best performance, with a responsivity enhancement of ~35 times and an improved on/off ratio of about 7 times compared to the un-imprinted device. The researchers attributed the high performance mainly to the nanopatterned grating structure, which helped improve the crystallinity of the film, optimized its nanostructure, enhanced carrier mobility, extended diffusion length, and improved photon absorption, ultimately enhancing the quantum efficiency of the device^133^. Shen et al. achieved efficient light capture by constructing an inverted pyramid structure on a silicon substrate. This structure significantly reduced the reflection losses of incident light, thereby improving light absorption efficiency. The calculated weighted average reflectance (*WAR*) decreased from 41.70% to 10.65%, indicating that the pyramid-structured substrate effectively enhanced the absorption of incident laser light. The calculation formula is as follows:

$${WAR}=\frac{{\int }_{400}^{1100}R\left(\lambda \right)N\left(\lambda \right)d\left(\lambda \right)}{{\int }_{400}^{1100}N\left(\lambda \right)d\left(\lambda \right)}$$

where *R(λ)* and *N(λ)* are the total reflectance and solar flux at Air Mass 1.5, respectively.

In addition, they simulated the electric field distribution of different structures using the finite-difference time-domain (FDTD) method. The results showed that, compared to a flat silicon substrate, the silicon substrate with an inverted pyramid structure exhibited a higher energy distribution, indicating that the patterned nanostructure design effectively enhanced the optical field and improved the optoelectronic detection performance^134^.

In addition to reducing reflection losses, patterning techniques can also enhance the optical resonance of optoelectronic films by constructing periodic micro-nano structures, such as nanopores, nanocolumns, and gratings, thereby improving light absorption efficiency and boosting the device’s quantum efficiency. Among them, localized surface plasmon resonance (LSPR) is a common method used to enhance the performance of optoelectronic detectors through microstructure. LSPR is an optical phenomenon that involves the localized focusing of the optical field near the surface of metallic plasmonic nanostructures, thereby enhancing the interaction between light and the material. This effect can significantly improve light absorption and has wide applications in nanophotonics, sensor technology, and optics. Xiao et al. constructed a high-performance photodetector by transferring multilayer InSe flakes onto a gold nanoparticle (Au NP) array and combining vacuum deposition of In thin films with in situ surface modification techniques. Experimental analysis indicated that the enhanced performance mechanism primarily results from the synergistic effect of two phenomena. Firstly, the LSPR effect in Au NP enhances light absorption, generating more high-energy free electrons. These thermally excited electrons can overcome the potential barrier and inject into the conduction band of InSe, thereby improving the effective utilization of charge carriers. Additionally, LSPR also excites a localized electromagnetic field through surface plasmon resonance, improving the energy conversion efficiency between light and the metal electrodes, further optimizing the optoelectronic performance of the PD. To further investigate the impact of LSPR on the detector’s performance, the researchers used the FDTD method to simulate the localized electric field distribution of Au NP with different sizes. The experimental results show that as the size of the Au NP increases from 8.7 nm to 46.8 nm, the maximum localized electric field intensity increases from 1.91 to 13.2, and then decreases to 8.7. Notably, when the diameter of the Au NP is 100 nm and the height is 23 nm, the localized electric field intensity is significantly enhanced, exhibiting a superior LSPR effect compared to nanoparticles of other sizes. The simulation results further confirm that Au NP with a diameter of 100 nm and a height of 23 nm significantly enhance the localized surface plasmon resonance, thereby improving the optoelectronic performance of the PD^135^. In addition, the fabrication of grating structures using patterning techniques can also effectively regulate the performance of PD. Cao et al. used a femtosecond laser system to fabricate a series of grating structures with different periods (Λ = 30, 20, 15, 14, 13, 12, and 10 µm), constructing patterned perovskite films with varying resolutions through laser etching, and systematically studied their optical properties. The study found that the fluorescence enhancement effect is highly dependent on the period and depth of the grating. Based on the fluorescence enhancement observed in the triangular grating structure of FAPbI_3_ perovskite, the researchers further fabricated high-performance perovskite PD. Compared to the unpatterned film devices, the PD exhibited a 6-fold increase in the on/off ratio, and its responsivity and detectivity were enhanced by 3 times and 12 times, respectively. Although the study did not provide a quantitative analysis of the PD performance for different grating period structures, the experimental results demonstrated that the structured films significantly improved device performance compared to conventional uniform films. Additionally, the enhancement effect of the PD varied with different grating periods, further proving the critical role of the grating period in optimizing PD performance^136^. Enhancing light absorption and optimizing the interaction between light and the PD film material through rational patterning structure design helps improve the device’s light absorption efficiency and quantum efficiency. During the device fabrication process, selecting the appropriate patterning method enables the precise design and fabrication of various surface structures, thereby effectively improving the performance of the PD.

The detectivity of PD is typically closely related to the responsivity and noise levels, while noise and response speed are, to some extent, influenced by the carrier transport and recombination processes, which are closely related to factors such as defect density in the material. The impact of patterning techniques on detectivity is primarily reflected in the modulation of the PD’s responsivity and noise characteristics. A well-designed patterning approach not only helps improve the crystallinity of the thin film but also effectively reduces interface scattering, enhances carrier mobility, thereby reducing noise and improving responsivity. Wang et al. employed plasma-enhanced chemical vapor deposition technology in combination with a mask template to fabricate in situ patterned 3D graphene/Si Schottky junction PD. The study results indicate that, compared to the unpatterned 3D graphene/Si Schottky PD, the patterned structure effectively reduces the dark current. In addition, after characterizing the noise current of both devices, it was found that the noise current of the patterned device was significantly reduced. This optimized carrier transport path and the suppression of electron-hole recombination help reduce dark current and noise current, thereby improving the overall performance and stability of the PD^137^. Hybrid perovskite crystals typically grow along the direction with the fastest growth rate under geometric constraints, resulting in the formation of large-sized grains. Inspired by this, Ding et al. employed a template-confined patterning method to fabricate MAPbCl_3_ perovskite thin films and PD with excellent response to linearly polarized light. The research results show that the template-confined growth patterning technique can induce the MAPbCl_3_ thin films to form highly oriented single-crystal structures, thereby promoting the formation of large grains and high crystallinity films. This optimized crystallization characteristic effectively reduces carrier recombination probabilities in the device and enhances carrier transport rates. In addition, the patterned structure enhanced the light absorption ability of the perovskite, further improving the detector’s light collection efficiency. As a result, the MAPbCl_3_ PD fabricated using the template method exhibited more stable photocurrents, with a dark current as low as 1.56 × 10^−11^ A, a detectivity of up to 3 × 10^12^ Jones, and a response time in the microsecond range^138^. Qiu et al. reported an ultra-compact near-infrared graphene PD based on a configurable 2D potential well. The PD utilizes photolithography combined with plasma etching to pattern the surface graphene layer, optimizing its optoelectronic response characteristics. Under illumination, photogenerated carriers in the graphene surface are effectively separated under the influence of the potential distribution. Electrons are confined to the low potential region, while holes migrate toward the high potential region, thereby enhancing the photogating effect and achieving optoelectronic gain. In addition, the 2D potential well-constructed by the dielectric structure exerts strong spatial confinement on the photogenerated carriers both laterally and vertically, effectively suppressing carrier recombination, thereby improving the device’s *EQE* and photonic gain^139^. In contrast, if the patterning process damages the film interface or leads to the accumulation of residues such as photoresist, which in turn increases the number of interface traps, the dark current of the device will increase, the responsivity will decrease, and the noise level will rise, thereby reducing the detectivity.

The defect concentration has a significant impact on the performance of PD. To construct high-performance PD, it is essential to ensure a low internal defect concentration in the material, along with efficient light absorption and conversion capabilities. While there is a lack of direct studies examining the relationship between patterning, defect concentration, and PD performance, existing research has explored the impact of defect concentration on PD characteristics. These studies suggest that lower defect concentrations generally lead to improved carrier mobility, reduced carrier recombination, and consequently enhanced optoelectronic performance, including responsivity, detectivity, and quantum efficiency. Yu et al. successfully synthesized three different compositions of perovskite single-crystal thin films (SCTF) using a template-confined patterning method, specifically FA_0.79_MA_0.13_Cs_0.08_, FA_0.82_MA_0.11_Cs_0.07_, and FA_0.85_MA_0.10_Cs_0.05_. Conventional characterization results show that the FA_0.82_MA_0.11_Cs_0.07_ SCTF, with the thinnest thickness of 1.62 µm, exhibits the highest trap-free hole mobility and the lowest surface defect density (2.29 × 10^9^ cm^-2^), indicating that this film possesses superior surface crystal quality and demonstrates good potential for optoelectronic detection applications. Additionally, compared to other previously reported non-integrated Cs-containing mixed cation perovskite PD, the PD based on the planar Au/FA_0.79_MA_0.13_Cs_0.08_ SCTF/Au structure achieved a *R* of 229.5 A W^−1^ and an *EQE* of 5.4 × 10^4^% under an incident light power of 0.02 mW cm^−2^ ^140^. These results demonstrate that by adjusting lattice strain and reducing surface defects, the responsivity and quantum efficiency of the PD can be significantly enhanced. When employing patterning techniques to fabricate PD, it is crucial to refine the process to prevent the introduction of defects or other unfavorable factors into the material films, thus ensuring the high performance of the device. The impact of patterning on device performance primarily manifests in aspects such as material defect density, crystallinity, and structural optimization. The selection of different patterning methods in device fabrication must comprehensively consider the material compatibility and the specific structural requirements of the target device to achieve optimal performance.

### Zero-dimensional perovskite photodetectors

0D perovskite materials, represented by perovskite NCs and perovskite QDs, not only have the inherent advantage of the high carrier mobility typical of perovskite materials but also have significantly high photoluminescence quantum yields and large optical absorption coefficients. These advantages have prompted numerous researchers to explore the uses of 0D patterned perovskite materials in PDs.

**Fig. 14:**
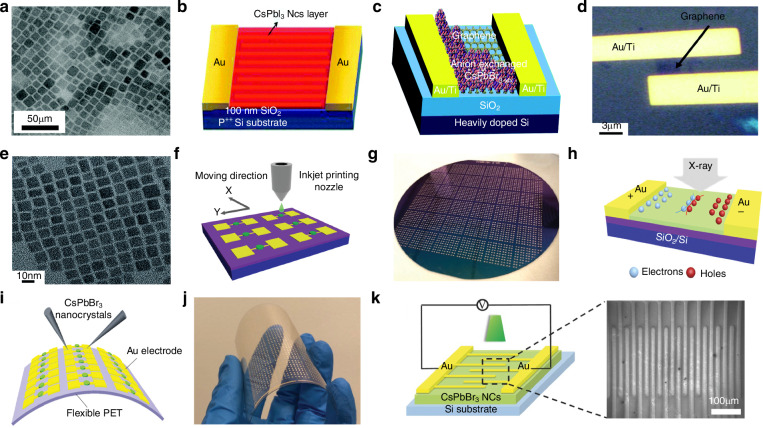
Perovskite nanocrystal photodetectors and device fabrication. **a** Transmission electron microscope (TEM) images of CsPbI_3_ NCs. Reproduced with permission^122^, Copyright 2016, Royal Society of Chemistry. **b** Schematic of the CsPbI_3_ NCs PD (*L* = 3 μm, *W* = 7800 μm). Reproduced with permission^122^, Copyright 2016, Royal Society of Chemistry. **c** Schematic of graphene–CsPbBr_3-x_I_x_ NCs PD. Reproduced with permission^141^, Copyright 2016, Royal Society of Chemistry. **d** Optical image of the graphene device. Reproduced with permission^141^, Copyright 2016, Royal Society of Chemistry. **e** TEM image of anion exchanged CsPbBr_3-x_I_x_ NCs. Reproduced with permission^141^, Copyright 2016, Royal Society of Chemistry. **f** Schematic of the key fabrication procedures for perovskite-based devices produced by inkjet printing. Reproduced with permission^128^, Copyright 2019 WILEY-VCH Verlag GmbH & Co. KGaA, Weinheim. **g** Photography of X-ray detector arrays on 4-inch wafer. Reproduced with permission^128^, Copyright 2019 WILEY-VCH Verlag GmbH & Co. KGaA, Weinheim. **h** Schematic of the configuration and working principle of the X-ray detector. Reproduced with permission^128^, Copyright 2019 WILEY-VCH Verlag GmbH & Co. KGaA, Weinheim. **i** Schematic of flexible perovskite-based X-ray detector arrays on a PET substrate. Reproduced with permission^128^, Copyright 2019 WILEY-VCH Verlag GmbH & Co. KGaA, Weinheim. **j** Photograph of a flexible device under bending. Reproduced with permission^128^, Copyright 2019 WILEY-VCH Verlag GmbH & Co. KGaA, Weinheim. **k** Schematic of the CsPbBr_3_ NC PD device structure and a microscope image of interdigitated electrodes with a finger width of 10 μm. Reproduced with permission^143^, Copyright 2019 WILEY-VCH Verlag GmbH & Co. KGaA, Weinheim

In 2016, Lim et al. first reported a method for constructing inorganic perovskite PDs based on patterned CsPbI_3_ perovskite NC films (Fig. 14a, b)^122^. The PD device had a photo-to-dark current ratio of up to 10^5^, with photocurrent rise and decay times of 24 ms and 29 ms, respectively. They also considered using carbon materials to improve the performance of 0D patterned perovskite PDs, presenting a construction scheme for a PD based on graphene-CsPbBr_3-x_I_x_ patterned NCs (Fig. 14d, e)^141^. The device structure is shown in Fig. 14c. After incorporating the graphene, the PD had a high photosensitivity. Under illumination with a wavelength of 405 nm and an intensity of 0.07 μW, it achieved a *R* of 8.2 × 10^8 ^A W^−1^, while the detection rate reached 10^16^ Jones.

High-performance PDs can be developed by combining perovskite materials with other materials and fully leveraging their respective advantages. Austin et al. employed an innovative approach by using CsPbX_3_ (where X is Br or I) perovskite NCs to improve the performance of graphene PDs. They designed a configuration in which these perovskite NCs act as the light-sensitive layer in graphene PDs, optimizing schemes for devices based on single-layer graphene (SLG) as well as inkjet-printed graphene (iG)^142^. They incorporated 5 mg mL^−1^ of perovskite NCs into a mixture of hexane, cyclohexanone, and terpineol (in 1:3:1 volume ratios) to create ink for inkjet printing. They then printed on SLG and inkjet-printed graphene to fabricate CsPb(Br/I)_3_/iG and CsPbBr_3_/SLG patterned perovskite PD devices. The PDs produced by this method had a high light responsivity of *R* > 10^6 ^A W^−1^ in the visible light wavelength range. A totally inkjet-printed PD achieved a responsivity of ~20 A W^−1^, representing the highest performance reported for such devices to date.

The use of inkjet printing in the construction of 1D patterned perovskite PDs extends beyond this, as Shabbir et al. reported a method for fabricating flexible X-ray PDs using high-quality colloidal CsPbBr_3_ perovskite QD by inkjet printing (Fig. 14f, g)^128^. They pre-deposited Au electrodes on SiO_2_/Si or flexible PET substrates using photolithography and electron beam evaporation, and then printed a CsPbBr_3_ QD solution onto the substrate by inkjet printing to fabricate large-area X-ray detector arrays. The resulting Au/CsPbBr_3_/Au patterned continuous perovskite thin film had a surface roughness of ~4 nm (Fig. 14h, i, j). The fabricated PDs demonstrated an exceptional sensitivity of up to 1450 µC Gy_air_^−1^ cm^−2^ at a bias voltage of 0.1 V, and were capable of detecting extremely low X-ray dose rates (≈17.2 µGy_air_ s^−1^). The device was very durable, with only a 12% decrease in current after 200 bend cycles.

The high carrier mobility, long carrier diffusion length, and excellent visible light absorption of perovskite materials can be combined with the localized surface plasmon resonance of noble metal NCs to have the advantages of both in the construction of 0D PDs. Zeng et al. designed a method for fabricating low-cost PDs by utilizing a solution made from CsPbBr_3_ perovskite and Au NCs, which was assembled into high-quality films by centrifugal casting and spin coating, followed by laser patterning (Fig. 14k)^143^. They dispersed 0D CsPbBr_3_ NCs in toluene to fabricate a patterned PD, which had a high on/off ratio exceeding 1.6 × 10^5^ under 532 nm laser illumination (4.65 mW cm^-2^) and a 2 V bias. They also introduced Au NCs to improve the device performance, resulting in a PD with a better sensitivity, achieving an on/off ratio greater than 10^6^ at 2 V bias. At the same time, the photocurrent increased from 245.6 μA to 831.1 μA, to give an enhancement factor of 238%, reaching the best values ever reported. After 10,000 light switching cycles, the 0D PD showed no significant degradation, demonstrating its excellent stability. Liu et al. demonstrated a method for fabricating patterned Cs_2_AgBiBr_6_ lead-free perovskites using a template-confined growth method and constructing a metal-semiconductor-metal perovskite PD, with TiN NC incorporated into the PD, which exhibited strong responsiveness in the near-infrared region^144^. When the device was irradiated with 850 nm light, the *R* reached 3.8 mA W^−1^, while the D* exceeded 10^9^ Jones, demonstrating excellent infrared detection sensitivity.

**Fig. 15:**
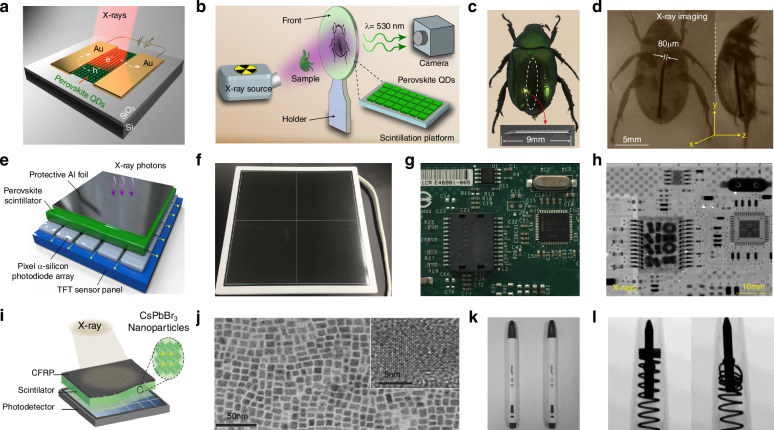
Perovskite QDs for X-ray imaging and scintillator applications. **a** Schematic showing the basic design of a perovskite-nanocrystal-based photoconductor used for X-ray sensing. Reproduced with permission^145^, Copyright 2018, Springer Nature Limited. **b** Schematic of the experimental setup used for real-time X-ray diagnostic imaging of biological samples. A beetle was placed between the X-ray source and a scintillation platform covered with perovskite QDs. Reproduced with permission^145^, Copyright 2018, Springer Nature Limited. **c** Bright-field images of the sample. Reproduced with permission^145^, Copyright 2018, Springer Nature Limited. **d** X-ray images of the sample, recorded at a voltage of 50 kV. Reproduced with permission^145^, Copyright 2018, Springer Nature Limited. **e** Multilayer design of the flat-panel X-ray imaging system. Reproduced with permission^145^, Copyright 2018, Springer Nature Limited. **f** Photograph of the packaged flat-panel detector. Reproduced with permission^145^, Copyright 2018, Springer Nature Limited. **g** Photograph of a network interface card. Reproduced with permission^145^, Copyright 2018, Springer Nature Limited. **h** X-ray image obtained using the flat-panel detector of a network interface card. Reproduced with permission^145^, Copyright 2018, Springer Nature Limited. **i** Schematic structure of a CsPbBr_3_ PNC scintillator based X-ray detector. Reproduced with permission^146^, Copyright 2018, WILEY-VCH Verlag GmbH & Co. KGaA, Weinheim. **j** TEM images of CsPbBr_3_ PNCs, the inset is a magnified image of a single CsPbBr_3_ PNC. Reproduced with permission^146^, Copyright 2018, WILEY-VCH Verlag GmbH & Co. KGaA, Weinheim. **k** Photograph of normal (left) and defective (right) ball-point pens with same exterior feature. Reproduced with permission^146^, Copyright 2018, WILEY-VCH Verlag GmbH & Co. KGaA, Weinheim. **l** X-ray images taken by a CsPbBr_3_ PNC scintillator based X-ray detector. Reproduced with permission^146^, Copyright 2018, WILEY-VCH Verlag GmbH & Co. KGaA, Weinheim

In the construction of perovskite PDs, 0D materials are frequently used used as downshifting materials in image sensors, specifically as scintillators. The scintillators enable the conversion of X-rays into visible light, facilitating X-ray imaging. In 2018 Yang et al. first reported the method of using CsPbX_3_ (where X is Cl, Br, or I) patterned perovskite NCs as scintillators to construct X-ray PDs (Fig. 15a, b, e)^145^. Since then, research on 0D patterned perovskite materials as scintillators has gained significant momentum (Fig. 15c, d, f, g, h). Im et al. designed a scheme for constructing X-ray detectors using CsPbBr_3_ patterned perovskite NCs^146^, as shown in Fig. 15i, j. In subsequent performance evaluations, the device showed an extremely fast response time of ~200 ns, significantly outperforming the current widely used terbium-doped gadolinium oxide X-ray PDs. To clearly demonstrate the imaging capabilities of the PD, they used two ballpoint pens, one with a functioning spring and one with a faulty spring, for X-ray imaging, as shown in Fig. 15k, l. Both the intact and damaged springs were distinctly identifiable, indicating the significant potential of 0D patterned perovskite scintillators for constructing an X-ray PD. While 0D patterned perovskite materials are often mixed with other semiconductors for PD applications, pure 0D patterned perovskite PDs still require more in-depth research.

### One-dimensional perovskite photodetectors

Compared to 0D perovskite PDs, 1D PDs with a high aspect ratio have several advantages, such as a larger volume ratio, easier surface functionalization, and more direct charge transport pathways. 1D perovskite materials are also very flexible, and because of this have been widely researched. When constructing patterned perovskite PDs, the overlap of 1D NW significantly impacts device performance, and current research is of into two main types: NW cross-linked networks and NW parallel arrays.

**Fig. 16:**
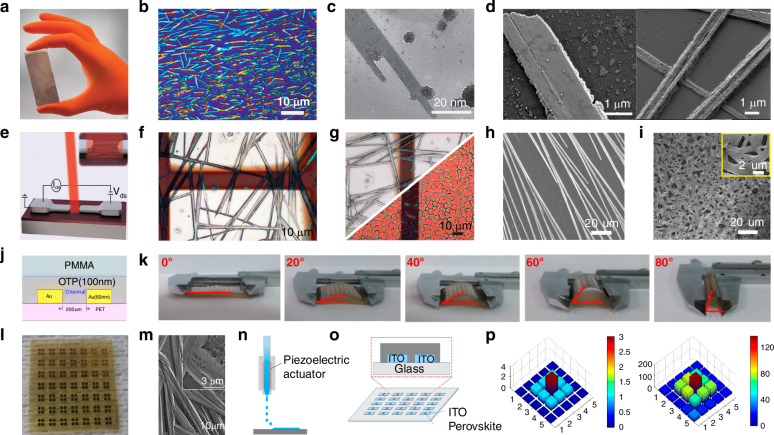
Perovskite nanowires and networks for photodetector applications. **a** Photo of the dimethylformamide coating formed on a glass microscope slide. Reproduced with permission^114^, Copyright 2014, American Chemical Society. **b** Optical microscope image of filiform crystallites grown on a SiO_2_/Si substrate. Reproduced with permission^114^, Copyright 2014, American Chemical Society. **c** TEM image of MAPbI_3_. Reproduced with permission^114^, Copyright 2014, American Chemical Society. **d** SEM images of micrometer-sized MAPbI_3_ filaments grown on a SiO_2_/Si surface. Reproduced with permission^114^, Copyright 2014, American Chemical Society. **e** Schematic of a nanowire-based device fabricated for FET. Reproduced with permission^114^, Copyright 2014, American Chemical Society. **f** Optical microscope image showing MAPbI_3_ nanowires crossing the Pt source-drain contacts deposited by electron beam evaporation. Reproduced with permission^114^, Copyright 2014, American Chemical Society. **g** Combined SEM-optical micrographs showing the surface of the thin film composed of nearly isotropic MAPbI_3_ particles (bottom) and nanowires (top) with the Pt source-drain contacts deposited by electron beam evaporation. Reproduced with permission^114^, Copyright 2014, American Chemical Society. **h** Perovskite morphologies from nanowires. Reproduced with permission^116^, Copyright 2015, American Chemical Society. **i** Perovskite morphologies from networks, the inset is a high-magnification image. Reproduced with permission^116^, Copyright 2015, American Chemical Society. **j** Schematic of a PD. Reproduced with permission^116^, Copyright 2015, American Chemical Society. **k** Photographs of a bent PD array at angles from 0° to 80°. Reproduced with permission^116^, Copyright 2015, American Chemical Society. **l** Photograph of a network PD array. Reproduced with permission^116^, Copyright 2015, American Chemical Society. **m** SEM image showing different perovskite morphologies produced by changing the substrate temperature to 35 °C. Reproduced with permission^84^, Copyright 2017, American Chemical Society. **n** Schematic showing inkjet printing on an ITO substrate. Reproduced with permission^84^, Copyright 2017, American Chemical Society. **o** Schematic of a PD array consisting of 25 pixels and the electrode-gap-electrode lateral structure of a single pixel. Reproduced with permission^84^, Copyright 2017, American Chemical Society. **p** Spatial distribution map of light power intensity and photocurrent output map calculated from the results of each pixel. Reproduced with permission^84^, Copyright 2017, American Chemical Society

In the study of PDs based on perovskite NW networks, Horváth et al. reported a detector based on a 1D perovskite NW cross-linked network, which involved placing MAPbI_3_ perovskite NW onto a silicon substrate with Pt contacts to fabricate the PD (Fig. 16a)^114^. The width of the NW used varied between 50 to 200 nm, with lengths up to 16 μm (Fig. 16b, c, d). Due to the challenges in precisely controlling the morphology and positioning of the perovskite NW (Fig. 16e, f, g), the device’s *R* was only 5 mA W^−1^, which is approximately four orders of magnitude lower than that of the best-performing PDs made from graphene and monolayer MoS_2_.

Developing controlled growth techniques for 1D perovskites is vital for investigating their use as PDs. Song et al. proposed a method of fabricating patterned MAPbI_3_ perovskite microwire networks and PDs on PET substrates using template-confined growth (Fig. 16h, i, j)^116^. The fabricated PD array had a *R* of 0.1 A W^−1^ and a D* of 1.02 × 10^12^ Jones, with a switching ratio of 300 and a response time as low as 0.3 ms. The patterned network also effectively distributed the stress applied to it, demonstrating its remarkable potential for use as flexible PD devices, because the photocurrent decreased by less than 10% after 10,000 bend cycles at angles not exceeding 60° (Fig. 16k, l).

Coincidentally, the inkjet printing method, which combines the benefits of maskless processing and easier control over patterning, has also been applied in the study of controlled growth for 1D perovskite materials (Fig. 16m, n)^84^. Li et al. used inkjet printing to fabricate a 5 × 5-pixel patterned MAPbI_3_ perovskite MW array (Fig. 16o). The devices had an excellent uniformity and a low defect density. At room temperature and a light power density of only 0.1 mW cm^−2^, the patterned perovskite micro-wire PD array had a high on/off ratio of up to 160, a *R* of 1.2 A W^−1^, and a D* of 2.39 × 10^12^ Jones, significantly outperforming the performance of spin-coated devices prepared under equivalent conditions (Fig. 16p).

**Fig. 17:**
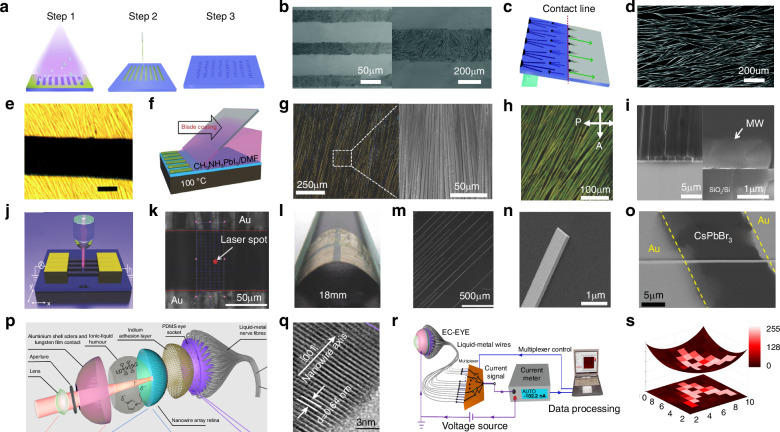
Growth and device integration of large-area perovskite nanowire/microwire arrays. **a** Schematic of selected area growth. Reproduced with permission^148^, Copyright 2015, Royal Society of Chemistry. **b** SEM images from three neighboring selected areas and one integrated area. Reproduced with permission^148^, Copyright 2015, Royal Society of Chemistry. **c** Schematic of aligned NW growth. The evaporation direction is indicated by arrows. Reproduced with permission^148^, Copyright 2015, Royal Society of Chemistry. **d** Optical images of NW grown by a modified evaporation-induced self-assembly method. Reproduced with permission^148^, Copyright 2015, Royal Society of Chemistry. **e** Optical image of a typical PD with a scale bar of 200 μm. Reproduced with permission^148^, Copyright 2015, Royal Society of Chemistry. **f** Illustration of the one-step blade coating process for the fabrication of single-crystal MAPbI_3_ MW arrays. Reproduced with permission^149^, Copyright 2016, WILEY-VCH Verlag GmbH & Co. KGaA, Weinheim. **g** Optical microscope and SEM images of the MW arrays. Reproduced with permission^149^, Copyright 2016, WILEY-VCH Verlag GmbH & Co. KGaA, Weinheim. **h** Cross-polarized optical image of the MW arrays. Reproduced with permission^149^, Copyright 2016, WILEY-VCH Verlag GmbH & Co. KGaA, Weinheim. **i** Cross-sectional SEM image of the MW arrays and a magnified SEM image. Reproduced with permission^149^, Copyright 2016, WILEY-VCH Verlag GmbH & Co. KGaA, Weinheim. **j** Measurement setup for scanning polycarbonate characterization. Reproduced with permission^149^, Copyright 2016, WILEY-VCH Verlag GmbH & Co. KGaA, Weinheim. **k** Optical microscope image of the scanning area in the device. The size of the laser spot is indicated by the red point. Reproduced with permission^149^, Copyright 2016, WILEY-VCH Verlag GmbH & Co. KGaA, Weinheim. **l** Photographs of the device when bent. Reproduced with permission^149^, Copyright 2016, WILEY-VCH Verlag GmbH & Co. KGaA, Weinheim. **m** SEM images of directional CsPbBr_3_ NW growth along faceted M-plane sapphire. Reproduced with permission^150^, Copyright 2017, American Chemical Society. **n** High-magnification SEM images of CsPbBr_3_ NW. Reproduced with permission^150^, Copyright 2017, American Chemical Society. **o** SEM image of the as-fabricated device. Reproduced with permission^150^, Copyright 2017, American Chemical Society. **p** Exploded view of an electrochemical eye (EC-EYE). Reproduced with permission^65^, Copyright 2020, The Author(s), under exclusive license to Springer Nature Limited. **q** High-resolution transmission electron microscope image of a single-crystal perovskite nanowire. Reproduced with permission^65^, Copyright 2020, The Author(s), under exclusive license to Springer Nature Limited. **r** Schematic of the measurement setup. Reproduced with permission^65^, Copyright 2020, The Author(s), under exclusive license to Springer Nature Limited. **s** Reconstructed image (letter ‘A’) of EC-EYE and its projection on a flat plane. Reproduced with permission^65^, Copyright 2020, The Author(s), under exclusive license to Springer Nature Limited

Compared to interconnected NW networks, patterned arrays composed of parallel 1D perovskite NW/MW and PDs constructed from these arrays, have superior performance in device uniformity and reproducibility^147^. Song et al. utilized a templating method to fabricate organic lead iodide perovskite (OIP) nanowires and used them in the construction of patterned perovskite PDs (Fig. 17a)^148^. The patterned arrays constructed from NW absorbed over 90% of the light from ultraviolet to the entire visible spectrum, exhibiting broadband absorption (Fig. 17b, c, d). The fabricated patterned perovskite PDs had a response time of ~0.3 ms, a *R* of 1.3 A W^−1^, and a D* of 2.5 × 10^12^ Jones (Fig. 17e). Jie et al. improved the template method and developed a blade-coating technique for the preparation of single-crystal arrays of MAPbI_3_ perovskite MW (Fig. 17f)^149^. They subsequently deposited 100 nm Au electrodes onto the MAPbI_3_ MW array to fabricate a patterned PD (Fig. 17g, h, i), which had a minimum detectable light intensity of 1 μW cm^−2^, with a *R* of up to 13.5 A W^−1^ and a D* of 5.25 × 10^12^ Jones. They also integrated a 21 × 21 pixels array of MAPbI_3_ perovskite MW with a 1 × 1 cm^2^ PET substrate (Fig. 17j, k, l), and the resulting device had photo current rise and decay times of 80 μs and 240 μs, respectively, which are significantly shorter than the time resolution ability of the human eye (~42 ms).

The two-step vapor deposition method has also been widely used in the fabrication of high-quality 1D patterned perovskite NW arrays. Pan et al. reported a method for producing oriented, ultra-long patterned CsPbBr_3_ perovskite NW arrays on sapphire substrates using controlled vapor deposition, which were then used to construct PDs^150^. The highly crystalline NW fabricated by the two-step vapor deposition method can reach lengths of several millimeters (Fig. 17m, n). They deposited 55 nm Au electrodes on the NW to construct PDs and evaluated the device performance (Fig. 17o). Under a drain voltage of 3 V, the device had an outstanding performance, with a *R* of 4.4 × 10^3 ^A W^−1^, and rise and decay times of 252 and 300 μs, respectively, outperforming PDs based on monohalide perovskite nano/microstructures of similar specifications. Fan et al. reported a method for fabricating MASnI_3_ perovskite NW arrays using a template-confined growth patterning technique and constructing a near-infrared PD^151^. The detector exhibits a *R* of 0.47 A W^−1^ and a D* of 8.8 × 10^10^ Jones when illuminated by near-infrared light with an intensity of 1.1 mW cm^-2^.

Building on the achievements of vapor deposition methods for patterned perovskite fabrication, Fan et al. proposed an exciting application for 1D patterned perovskite NW arrays: an electrochemical eye. They demonstrated an electrochemical eye device composed of a hemispherical retina made from a high-density array of perovskite NW^65^. They fabricated the array using a two-step vapor deposition method (Fig. 17q). They also used liquid metal wires as contacts for the PD, creating a hemispherical PD retinal and then conducted performance evaluations of the device (Fig. 17p, r). The device exhibited rise and decay times of 19.2 ms and 23.9 ms, respectively, which are well below the response time of the human eye. The linear dynamic range was from 0.3 μW cm^−2^ to 50 mW cm^−2^, with a *R* of 303.2 mA W^−1^ and a D* of 1.1 × 10^9^ Jones. In addition to demonstrating excellent performance, the device showed no significant degradation after 64,800 cycles, indicating remarkable stability. The diagonal field of view of the eye was ~100.1°, surpassing the 69.8° diagonal field of view of planar devices (Fig. 17s), thereby highlighting the exciting possibility of using 1D patterned perovskite NW arrays in high-density integrated photonic devices.

The PDs based on 1D patterned perovskites have an impressive performance, including ultra-high responsivity. However, the inherent large surface area of 1D perovskites leads to a high density of electronic surface traps, and the presence of significant surface defects can adversely affect the performance and stability of the devices, severely limiting their practical applications.

### Two-dimensional perovskite photodetectors

The physicochemical properties of 2D perovskite materials highlight advantages such as tunable optical band gaps and high photoluminescence quantum yields^152,153^, drawing increased attention to the research on 2D patterned perovskite films in the field of PDs. There are two main approaches for constructing 2D patterned perovskite PDs: one involves thinning down pre-prepared 3D perovskite structures to create atomically thin 2D patterned perovskite films for device fabrication; the other entails directly synthesizing 2D patterned perovskite films for device construction.

**Fig. 18:**
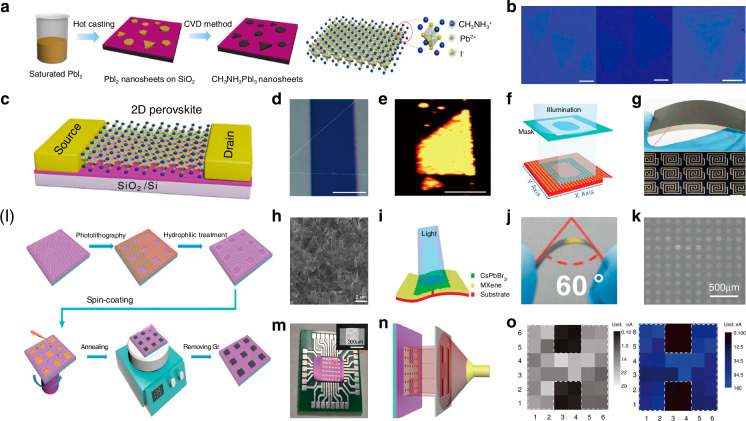
2D perovskite nanosheets and arrayed photodetector applications. **a** Schematic of the solution process to fabricate 2D PbI_2_ nanosheets and the vapor-phase conversion process to transfer PbI_2_ into 2D MAPbI_3_ perovskite nanosheets. Reproduced with permission^123^, Copyright 2016, American Chemical Society. **b** Left: High-magnification optical image of 2D MAPbI_3_ nanosheets. Scale bar: 4 μm. Right: Optical images of the as-grown MAPbI_3_ nanosheet with different thicknesses (thin MAPbI_3_ sheet with a thicker region at the center). Scale bar: 10 μm. Reproduced with permission^123^, Copyright 2016, American Chemical Society. **c** Schematic of a transistor device based on 2D perovskite. Reproduced with permission^123^, Copyright 2016, American Chemical Society. **d** Optical image of the 2D perovskite device. Scale bars: 10 μm. Reproduced with permission^123^, Copyright 2016, American Chemical Society. **e** photoluminescence map of the 2D perovskite device. Scale bars: 10 μm. Reproduced with permission^123^, Copyright 2016, American Chemical Society. **f** Schematic of a digital sensor based on a large-area array 1665-pixel PD. Reproduced with permission^117^. Copyright 2019 WILEY-VCH Verlag GmbH & Co. KGaA, Weinheim. **g** A large-area array MXene electrode and the corresponding microscopic pattern. Scale bar: 1 mm. Reproduced with permission^117^. Copyright 2019 WILEY-VCH Verlag GmbH & Co. KGaA, Weinheim. **h** SEM image of 2D CsPbBr_3_. Reproduced with permission^117^. Copyright 2019 WILEY-VCH Verlag GmbH & Co. KGaA, Weinheim. **i** Schematic of a single PD based on a MXene electrode. Reproduced with permission^117^. Copyright 2019 WILEY-VCH Verlag GmbH & Co. KGaA, Weinheim. **j** Image of the PDs at a 60° bending angle. Reproduced with permission^117^. Copyright 2019 WILEY-VCH Verlag GmbH & Co. KGaA, Weinheim. **k** SEM image of a perovskite film with a periodic square array pattern. Reproduced with permission^117^. Copyright 2019 WILEY-VCH Verlag GmbH & Co. KGaA, Weinheim. **l** Illustration of the procedures for preparing patterned perovskite films. Reproduced with permission^155^. Copyright 2019 WILEY-VCH Verlag GmbH & Co. KGaA, Weinheim. **m** Optical photograph of a typical 6 × 6 perovskite PD arrays fixed onto a printed circuit board, the inset shows an SEM image of a single pixel device. Reproduced with permission^155^. Copyright 2019 WILEY-VCH Verlag GmbH & Co. KGaA, Weinheim. **n** Schematic of the measurement setup for the perovskite PD arrays to realize visible light imaging sensing. Reproduced with permission^155^. Copyright 2019 WILEY-VCH Verlag GmbH & Co. KGaA, Weinheim. **o** Corresponding 2D current map of the PD arrays upon illumination with white light (350 µW cm^−2^) and blue light (450 nm, 1.45 mW cm^−2^). Reproduced with permission^155^. Copyright 2019 WILEY-VCH Verlag GmbH & Co. KGaA, Weinheim

A two-step vapor-phase process can produce 2D patterned MAPbI_3_ perovskite NCs. Zhang et al. fabricated MAPbI_3_ perovskite single-crystal nanosheets, constructed field-effect transistor PDs, and tested their optoelectronic performance (Fig. 18a–e)^123^. The PDs exhibited a *R* of 22 A W^−1^ under a bias voltage of 1 V and 405 nm laser illumination, which decreased to 12 A W^−1^ when the laser wavelength was changed to 532 nm, outperforming bulk perovskite film PDs (3 A W^−1^)^154^. Additionally, the rise and decay times of the photocurrent were less than 20 ms and 40 ms, respectively, demonstrating faster responses compared to bulk perovskite PDs.

Wang et al. fabricated a 2D patterned MAPbI_3_ perovskite array using a two-step vapor deposition method^46^. The dimensions of the array were ~10 µm, with a thickness ranging from 300 to 500 nm. They then used this method for the manufacture of patterned PDs, growing 2D perovskite crystals on electrodes. The perovskite initially nucleated at the hydrophilic sites between the two electrodes on the pre-patterned substrate and gradually extended towards the electrodes, forming a dual-probe PD structure. Performance measurements showed that the device had a *R* of ~7 A W^−1^, along with a high-speed photonic response time of 500 µs, demonstrating its potential for many applications.

Coincidentally, Yang et al. also proposed a method a PD construction based on the direct synthesis of 2D layers of a patterned CsPbBr_3_ perovskite^117^. Using inkjet printing, they first prepared a uniform MXene conductive film, followed by UV lithography to pattern coil-shaped electrodes on it (Fig. 18g, h). Finally, they spray-coated a solution of the 2D CsPbBr_3_ perovskite nanosheets onto the MXene electrodes to fabricate a flexible PD. Under a bias voltage of 10 V, the device had a *R* of 44.9 mA W^−1^ and a D* of 6.4 × 10^8^ Jones. They also conducted bending tests on the device, which retained 85% of its initial photocurrent characteristics after 1500 cycles of bending to 160°, demonstrating its excellent stability (Fig. 18i, j, k). As shown in Fig. 18f, they constructed a large-area sensor with 1665 pixels, covering 72 cm^2^, capable of clearly transmitting the “0” symbol for optical communication.

In addition to inkjet printing, template methods have been widely used for the direct synthesis of 2D patterned perovskite materials. Luo et al. used this technique with a graphene-assisted hydrophilic-hydrophobic surface to induce the growth of Cs-doped patterned FAPbI_3_ perovskite films and fabricate PDs (Fig. 18l)^155^. They prepared smooth perovskite films with a root mean square roughness of ~9.71 nm using the method, and then constructed an optical image sensor consisting of a 6 × 6 pixel array of 2D patterned perovskite film PDs (Fig. 18m). Under an incident light wavelength at 650 nm and illumination intensity of 23.1 µW cm^−2^, the sensor had a maximum *R* of up to 4.8 A W^−1^ and a peak D* of 4.2 × 10^12^ Jones. It also had rise and decay times of 13.7 µs and 14.9 µs, respectively, significantly faster than PDs based on perovskite networks (Fig. 18n, o). The minimal differences between the pixels of the device indicate its tremendous potential for use in large-scale integrated devices. Recently, Fang et al. reported a method for synthesizing surface-patterned NH_3_(CH_2_)_4_NH_3_PbBr_4_ perovskite 2D microdisks through a template-assisted patterning technique and constructed a PD^156^. The PD exhibits a significant optical response in the UV wavelength range, with an on/off ratio approaching 5000, a *R* of 2.24 A W^−1^, and a D* of 10^13^ Jones. Moreover, even under weak light illumination, the PD demonstrates excellent imaging capabilities. Similarly, Kim et al. also reported a method for fabricating patterned CsPbBr_3_ perovskite films based on seed-induced patterning^157^. They fabricated a pixel array of 10 × 30 μm^2^ on a PET substrate and constructed a PD. The device exhibited a switch-on/off ratio exceeding 10^3^ under 365 nm UV light, and showed no degradation in photoluminescence performance after 1000 bending cycles, demonstrating excellent mechanical stability.

**Fig. 19:**
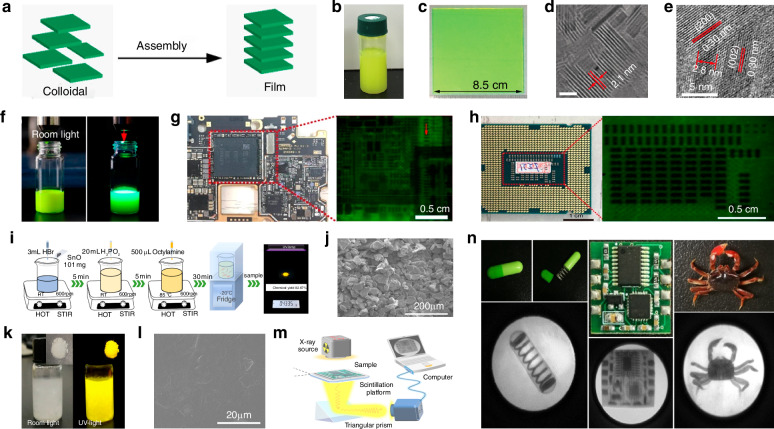
Perovskite nanosheets and layered perovskite scintillators for X-ray imaging. **a** Schematic showing the self-assembly of CsPbBr_3_ nanosheets. Reproduced with permission^158^, Copyright 2019, American Chemical Society. **b** Photograph of a heavily loaded colloid (150 mg mL^−1^) in toluene. Reproduced with permission^158^, Copyright 2019, American Chemical Society. **c** A wafer-size thin film on a glass slide. Reproduced with permission^158^, Copyright 2019, American Chemical Society. **d** TEM image of assembled CsPbBr_3_ nanoplatelets with an interplane spacing of 2.1 nm. Reproduced with permission^158^, Copyright 2019, American Chemical Society. **e** HRTEM image of (**d**), showing a clear lattice fringe of (200) facets. Reproduced with permission^158^, Copyright 2019, American Chemical Society. **f** Photographs of the concentrated colloid (0.15 g mL^−1^) under ambient light and X-rays. Reproduced with permission^158^, Copyright 2019, American Chemical Society. **g** Photograph of a transistor panel in a cellphone. The interior structure of the resin-covered panel (red dashed square) is clearly seen using a perovskite thin-film screen under X-radiation. Reproduced with permission^158^, Copyright 2019, American Chemical Society. **h** Photograph of a standard central processing unit panel with a silicon chip integrated underneath. X-ray image of the part covered (red dashed square) by the silicon chip, showing good resolution. Reproduced with permission^158^, Copyright 2019, American Chemical Society. **i** Schematic of the synthesis of 2D layered (C_8_H_17_NH_3_)_2_SnBr_4_ perovskite scintillators. Reproduced with permission^159^, Copyright 2020, American Chemical Society. **j** High-resolution TEM image of the prepared (C_8_H_17_NH_3_)_2_SnBr_4_ perovskite. Reproduced with permission^159^, Copyright 2020, American Chemical Society. **k** Photograph of a (C_8_H_17_NH_3_)_2_SnBr_4_ colloidal solution under ambient light (left) and UV light of 365 nm (right). Reproduced with permission^159^, Copyright 2020, American Chemical Society. **l** SEM image of the exterior surface of the perovskite scintillator film. Reproduced with permission^159^, Copyright 2020, American Chemical Society. **m** Schematic of the experimental setup used for X-ray imaging of samples. Reproduced with permission^159^, Copyright 2020, American Chemical Society. **n** Photographs of target materials (a capsule containing a spring inside, a circuit board, and a crab) and the images obtained by X-ray imaging. Reproduced with permission^159^, Copyright 2020, American Chemical Society

Similar to their 0D counterparts, 2D perovskite materials can also be utilized in X-ray image sensors as scintillator materials. Zhang et al. reported a method for synthesizing 2D patterned CsPbBr_3_ perovskite nanosheets at room temperature to create colloidal scintillators (Fig. 19a, b)^158^. The nanosheets were stacked face-to-face with an interlayer spacing of 2.1 nm, and a large-area film measuring 8.5 × 8.5 cm^2^ (Fig. 19c, d, e), was cast as a colloidal scintillator which was uniform and crack-free, and used as a high-resolution X-ray PD. They applied it to perform X-ray imaging of a smartphone panel, clearly revealing the detailed structural information of the transistor panel under the resin cover (Fig. 19f, g, h). In addition to 2D patterned perovskite nanosheets produced by a thinning down pre-prepared 3D perovskite approach being used as scintillators, directly synthesized materials have also been used. Fu et al. were the first to propose using 2D patterned (C_8_H1_7_NH_3_)_2_SnBr_4_ perovskite and perovskite scintillator films coated with a polymer layer in X-ray imaging systems (Fig. 19i, j)^159^. A scintillator based on this material has a high quantum yield of up to 98% and a long photoluminescence lifetime of 3.34 µs (Fig. 19k). They later developed an X-ray imaging scintillation platform measuring 100 × 100 mm^2^ (Fig. 19l). As shown in Fig. 19m, n, X-ray imaging was conducted on three samples: a capsule containing a spring, a circuit board, and a crab, all of which produced clear images, indicating the potential of directly synthesized 2D perovskite materials in the scintillator field.

2D patterned perovskite materials demonstrate advantages such as fewer surface defects and improved stability in practical applications. However, PDs based on 2D perovskite materials often have a relatively low responsivity which may be attributed to factors such as a large bandgap, low carrier mobility, and poor contact between the perovskite and the electrodes^160^. Future research on 2D patterned perovskite PDs needs to address these issues through the development of new materials and the design of device structures.

### Three-dimensional perovskite photodetectors

When constructing patterned perovskite PD devices, it is essential to consider how to maximize the advantages of the materials, such as a high charge carrier mobility, long charge carrier diffusion length, and excellent stability, to achieve a high detection performance. 3D perovskite materials have a low trap density and good stability^161^, making them popular for PD construction. The most commonly used 3D perovskite materials can be categorized into polycrystalline perovskite films and single-crystal perovskites.

**Fig. 20:**
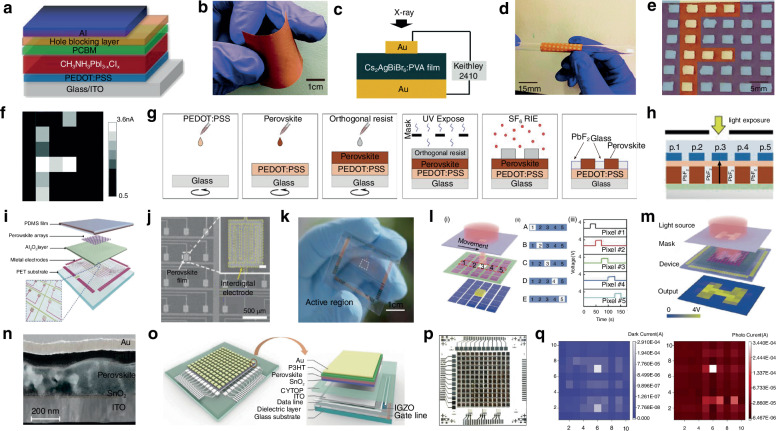
Perovskite photodetector arrays and flexible device integration. **a** Structure of the hybrid perovskite PD. Reproduced with permission^124^, Copyright 2014, Springer Nature Limited. **b** Optical photo of a 100 μm-thick Cs_2_AgBiBr_6_/PVA (2 : 1 weight ratio). Reproduced with permission^129^, Copyright 2018, Royal Society of Chemistry. **c** Schematic showing the structure of an X-ray detector with a Cs_2_AgBiBr_6_/PVA composite film as an X-ray photoconductor. Reproduced with permission^129^, Copyright 2018, Royal Society of Chemistry. **d** Photo showing an array of X-ray detectors fabricated on a 100 μm-thick Cs_2_AgBiBr_6_/PVA composite film. Reproduced with permission^129^, Copyright 2018, Royal Society of Chemistry. **e** Photo showing an X-ray imager with 6 × 6 detectors fabricated on a 100 μm-thick Cs_2_AgBiBr_6_/PVA composite film. Reproduced with permission^129^, Copyright 2018, Royal Society of Chemistry. **f** Photocurrent contrast between the pixels resolving the “F” pattern of a molybdenum foil. Reproduced with permission^129^, Copyright 2018, Royal Society of Chemistry. **g** Schematic of the photolithographic patterning of MAPbI_3_ films. Reproduced with permission^125^. Copyright 2016 WILEY-VCH Verlag GmbH & Co. KGaA, Weinheim. **h** Structure of an organic perovskite photodiode for patterned device. Reproduced with permission^125^. Copyright 2016 WILEY-VCH Verlag GmbH & Co. KGaA, Weinheim. **i** Schematic of the device structure. Inset: The design of a complex circuit. Reproduced with permission^115^. Copyright 2018 WILEY-VCH Verlag GmbH & Co. KGaA, Weinheim. **j** SEM image of a PD array and a magnified image of a single pixel in the device (scale bar is 20 µm). Reproduced with permission^115^. Copyright 2018 WILEY-VCH Verlag GmbH & Co. KGaA, Weinheim. **k** Optical image of 10 × 10 pixelated flexible PD arrays. Reproduced with permission^115^. Copyright 2018 WILEY-VCH Verlag GmbH & Co. KGaA, Weinheim. **l** Schematic of the light spot moving process. Reproduced with permission^115^. Copyright 2018 WILEY-VCH Verlag GmbH & Co. KGaA, Weinheim. **m** Schematic of a flexible PD array to detect multipoint light distribution. Reproduced with permission^115^. Copyright 2018 WILEY-VCH Verlag GmbH & Co. KGaA, Weinheim. **n** Cross-sectional TEM image of a (FASnI_3_)_0.6_(MAPbI_3_)_0.4_ perovskite PD. Reproduced with permission^162^. Copyright 2019 WILEY-VCH Verlag GmbH & Co. KGaA, Weinheim. **o** Schematic of a 12 × 12 matrix indium-gallium-zinc oxide (IGZO) thin film transistor (TFT) array and a single pixel of perovskite PDs integrated with IGZO TFTs. Reproduced with permission^162^. Copyright 2019 WILEY-VCH Verlag GmbH & Co. KGaA, Weinheim. **p** Photo of a 12 × 12 matrix IGZO TFT array. Reproduced with permission^162^. Copyright 2019 WILEY-VCH Verlag GmbH & Co. KGaA, Weinheim. **q** Dark and photocurrent maps of a 10 × 10 perovskite-based photodiode array under 850 nm incident light with 1374 mW cm^−2^ illumination biased at −0.1 V. Reproduced with permission^162^. Copyright 2019 WILEY-VCH Verlag GmbH & Co. KGaA, Weinheim

3D polycrystalline perovskite films were the first to be used in the construction of PDs. In 2014 Xie et al. reported a method for fabricating MAPbI_3_ polycrystalline perovskite films by spin-coating on ITO substrates and constructing a broadband PD^119^. They employed two adjacent ITO films as conductive electrodes and filled the space between them with a MAPbI_3_ film ~1.5 μm thick to construct the PD, which they subsequently tested for performance. The PD exhibited a wide spectral response range from 310 nm to 780 nm. Under a bias voltage of 3 V, the device had a *R* of 3.49 A W^−1^ and an external quantum efficiency of 1.19 × 10^3^% for light excitation at 365 nm. When the excitation wavelength was changed to 780 nm, the responsivity decreased to 0.0367 A W^−1^, with an external quantum efficiency of 5.84%. This demonstrates the significant potential of patterned 3D perovskite polycrystalline films in PD research. In the same year, Yang et al. designed a novel PD based on organic-inorganic hybrid perovskite materials^124^. The device had an ITO/TiO_2_/MAPbI_3-x_Cl_x_/p-doped spiro-MeOTAD/Au (PD4) structure (Fig. 20a). Operating at room temperature, it achieved a D* of up to 10^14^ Jones and a linear dynamic range exceeding 100 dB, significantly outperforming traditional inorganic semiconductor PDs at that time.

Since the first report of 3D patterned perovskite materials being used to construct PDs, improving device performance has become a key focus for researchers. Yu et al. designed a scheme using lead-free halide double perovskite and polymer composite films as X-ray photoconductors to construct patterned X-ray PDs^129^. They constructed the PD using Au films as both the top and bottom contact layers, with a Cs_2_AgBiBr_6_/PVA perovskite patterned composite film serving as the X-ray absorption layer (Fig. 20b, c, d). When a voltage of 400 V was applied to the device, the photocurrent reached ~10 nA, while the sensitivity achieved 40 μC Gy_air_^−1^ cm^−2^, matching the sensitivity levels of single-crystal Cs_2_AgBiBr_6_ perovskite PDs developed during the same period. They also constructed a pixelated X-ray imaging device with a 6 × 6 detector pixel array on a single composite film and conducted tests on it. As shown in Fig. 20e, f, the X-ray image of the letter “F” and patterns composed of pixels were clearly resolved, demonstrating the significant potential of 3D patterned perovskite polycrystalline films in the field of PDs.

In addition to the previous approach of constructing PDs on a continuous 3D patterned perovskite polycrystalline film, the idea of using a separated array of perovskite polycrystalline films to build PDs has also been proposed. Using advances in photolithography, Zakhidov et al. reported a method that employed hydrofluoroether solvents for photolithographic processing to develop high-resolution patterned MAPbI_3_ perovskite polycrystalline films and fabricate thin-film-based PDs (Fig. 20g, h)^125^. Using an orthogonal photolithography patterning method, they fabricated a patterned MAPbI_3_ perovskite thin film array on an ITO substrate with a resolution as low as 2 μm. They constructed a detector based on this array, that had a linear dynamic range of 80 dB and a power conversion efficiency of 11.7%. Importantly, non-patterned devices had a significant crosstalk of 96% between adjacent pixels, whereas those constructed by Zakhidov et al. using the separated perovskite polycrystalline film array reduced signal crosstalk to 21%, which indicates that the patterning process is crucial for minimizing crosstalk between pixels in such arrays.

In addition to lithography techniques, the two-step vapor phase method and seed-assisted growth method have found significant use in constructing PDs based on separated perovskite polycrystalline film arrays. Pan et al. proposed a scheme for fabricating 3D patterned MAPbI_3-x_Cl_x_ perovskite arrays using the two-step vapor phase method and for constructing PDs (Fig. 20i)^115^. They synthesized large patterned perovskite arrays on a PET substrate and deposited metal layers as electrodes to construct a large-scale flexible PD array (Fig. 20j, k). Under illumination at a wavelength of 650 nm and an intensity of 38.3 mW cm^−2^, the device exhibited a high on/off current ratio of up to 1.2 × 10^3^. At an illumination intensity of 0.033 mW cm^−2^, the *R* reached 2.17 A W^−1^, with a detection rate of 9.4 × 10^11^ Jones. They also demonstrated the performance of the PD using a flexible PD array with a resolution of 63.5 dots per inch, clearly recognizing the letter “H” as shown in Fig. 20l, m.

Zhou et al. reported a method for constructing Sn-Pb based perovskite PD arrays on an IGZO thin-film transistor matrix plane using a seed-induced growth technique (Fig. 20n)^162^. They constructed a 12 × 12-pixel PD matrix array and tested its performance (Fig. 20o, p). The device had a broad spectral response range from 300 to 1000 nm, achieving a D* of 10^11^ Jones under 850 nm wavelength illumination, with rise and decay times of 19 ms and 13 ms, respectively. They also demonstrated the device’s infrared imaging ability by illuminating it with infrared light at a wavelength of 850 nm and an intensity of ~1374 mW cm^−2^ through an “11” shaped mask. The result in Fig. 20q, clearly shows the number “11.”

During the investigation of 3D patterned perovskite PDs, it has been found that these single crystal materials have a superior optoelectronic performance to polycrystalline films, such as a higher carrier mobility, longer carrier diffusion length, and better stability.

**Fig. 21:**
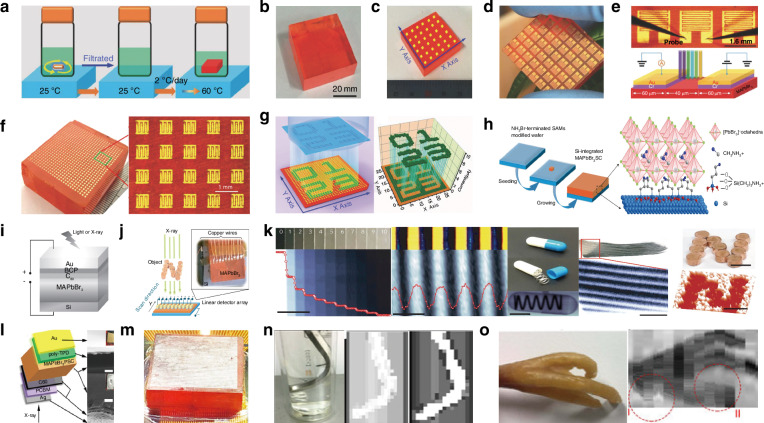
Perovskite single crystal growth and large-area image sensor arrays. **a** Schematic of low-temperature-gradient crystallization for the MAPbBr_3_ single crystals. Reproduced with permission^120^. Copyright 2018 WILEY-VCH Verlag GmbH & Co. KGaA, Weinheim. **b** Photograph taken from a MAPbBr_3_ single crystal grown using the low-temperature-gradient crystallization. Reproduced with permission^120^. Copyright 2018 WILEY-VCH Verlag GmbH & Co. KGaA, Weinheim. **c** Photo of a high-quality MAPbBr_3_ single crystal used for the PL uniformity measurement. Reproduced with permission^120^. Copyright 2018 WILEY-VCH Verlag GmbH & Co. KGaA, Weinheim. **d** Photo of 56 photosensors fabricated on a piece of MAPbBr_3_ single crystal. Reproduced with permission^120^. Copyright 2018 WILEY-VCH Verlag GmbH & Co. KGaA, Weinheim. **e** Schematic of the device operation. Reproduced with permission^120^. Copyright 2018 WILEY-VCH Verlag GmbH & Co. KGaA, Weinheim. **f** Photograph of a 27 × 27 photosensor array fabricated on a 34 × 38 mm^2^ MAPbBr_3_ single crystal. Reproduced with permission^120^. Copyright 2018 WILEY-VCH Verlag GmbH & Co. KGaA, Weinheim. **g** Schematic of the projection imaging mechanism. Reproduced with permission^120^. Copyright 2018 WILEY-VCH Verlag GmbH & Co. KGaA, Weinheim. **h** Schematic of the fabrication of Si-integrated MAPbBr_3_ single crystals. Reproduced with permission^163^. Copyright 2017, Springer Nature Limited. **i** Schematic of the structure of Si-integrated MAPbBr_3_ single crystal devices. Reproduced with permission^163^. Copyright 2017, Springer Nature Limited. **j** Schematic of X-ray imaging with Si-integrated MAPbBr_3_ single crystal detectors. Reproduced with permission^163^. Copyright 2017, Springer Nature Limited. **k** Optical (top) and X-ray (bottom) images of stacked glass coverslips, a stainless-steel plate with etched-through lines, an encapsulated metallic spring, a fish caudal fin and an ‘N’ copper logo. Reproduced with permission^163^. Copyright 2017, Springer Nature Limited. **l** Schematic of the device structure and a corresponding cross-sectional SEM image, both scale bars 500 nm. Reproduced with permission^130^. Copyright 2018 WILEY-VCH Verlag GmbH & Co. KGaA, Weinheim. **m** Photo of the PIN array. Reproduced with permission^130^. Copyright 2018 WILEY-VCH Verlag GmbH & Co. KGaA, Weinheim. **n** Photo and X-ray images using low/high photon energies. Reproduced with permission^130^. Copyright 2018 WILEY-VCH Verlag GmbH & Co. KGaA, Weinheim. **o** Optical image of a chicken claw and a corresponding low energy X-ray image. Reproduced with permission^130^. Copyright 2018 WILEY-VCH Verlag GmbH & Co. KGaA, Weinheim

Liu et al. proposed using the high carrier mobility of 3D patterned perovskite single crystal materials for fabricating patterned MAPbBr_3_ perovskite single crystals for PD applications using a liquid-phase approach (Fig. 21a)^120^. They synthesized MAPbBr_3_ perovskite single crystals with dimensions of 44 × 49 × 17 mm^3^ which had a high carrier mobility of 81 ± 5 cm^2^ Vs^−1^, while the trap concentration was also found to be relatively low, at 6.2 ± 2.7 × 10^9 ^cm^−3^ (Fig. 21b, c). They then constructed a patterned Au/MAPbBr_3_ (2.6 mm)/Au perovskite PD and tested it (Fig. 21d, e) under 525 nm wavelength illumination and a bias voltage of 4 V. The device had a *R* of up to 1.6 × 10^4 ^mA W^−1^, a D* of up to 6 × 10^13^ Jones, an external quantum efficiency of 3900%, and a linear dynamic range of 81 dB, outperforming silicon-based PDs. To more clearly demonstrate the performance of the 3D patterned perovskite single-crystal PD, they constructed a large-area PD array with 729 pixels on ~1300 mm^2^ of perovskite single-crystal material. As shown in Fig. 21f, g, when optical patterns were projected onto the array after passing through a light-shielding mask, the three displayed patterns-numbers, letters, and images-were all clearly discernible.

We previously introduced the approach of constructing X-ray PDs using patterned perovskites by thinning 3D structures to 2D. Currently, the direct use of 3D patterned perovskite materials for the construction of X-ray PDs is also a focus of ongoing research. Huang et al. reported a method for fabricating patterned MAPbBr_3_ perovskite single crystals on silicon substrates, which serves as the basis for constructing X-ray PDs^163^. Using the liquid-phase method shown in Fig. 21h, i, j, they integrated patterned MAPbBr_3_ perovskite single crystals on a silicon substrate and constructed silicon-integrated MAPbBr_3_ single crystal X-ray PD devices. The devices exhibited an extremely high X-ray sensitivity of up to 21 µC/mGy_air_ cm^2^, which is ~1000 times that of commercially available amorphous selenium-based X-ray detectors. To visually demonstrate the performance of the X-ray PD, they used it to X-ray image a capsule containing a metal spring which is opaque to visible light. As shown in Fig. 21k, the spring in the capsule is clearly visible, and the variations in the thickness of the capsule itself can even be discerned, highlighting the significant potential of the PD in medical imaging applications. Coincidentally, Chen et al. also proposed a scheme for constructing an X-ray PIN diode based on 3D patterned MAPbBr_3_ perovskite single crystals^130^. The device structure is shown in Fig. 21l. When subjected to light at a wavelength of 532 nm and an intensity of 1 mW cm^−2^, the device had a *R* of 1.252 mA W^−1^, while its X-ray sensitivity reached 23.6 µC mGy_air_^−1^ cm^−2^. Subsequently, they constructed a 40 × 40 patterned MAPbBr_3_ perovskite PIN diode PD array (Fig. 21m), which was used to perform X-ray imaging of a metal rod submerged in a cup of water and a chicken foot. As seen in Fig. 21n, o, the metal rod was clearly discernible, and the fibula and radius of the chicken foot were also distinctly identifiable, demonstrating the immense potential of 3D patterned perovskite single crystal PDs.

As shown in Table 1, we summarized and compared the PD performance presented in the articles. 0D perovskite structures (such as quantum dots and nanocrystals) exhibit high sensitivity and broad spectral response capabilities in optoelectronic detection due to their tunable size, high extinction coefficients, and multiple exciton generation properties. PD based on CsPbBr_3_ NC have achieved a switching ratio exceeding 10^6^ ^143^. However, 0D materials face issues such as surface defects and size uniformity. Future improvements can be made by optimizing surface states through ligand engineering and in situ passivation techniques, combined with inkjet printing patterning technology and photolithography patterning technology to achieve the integration of high-resolution devices. 1D perovskite structures (such as nanowires and nanorods) exhibit exceptional performance in polarization light detection due to their high crystal quality, anisotropic geometry, and long carrier lifetime. The PD constructed based on patterned MAPbI_3_ nanowire networks achieves a detection rate of 1.02 × 10^12^ Jones^116^. However, 1D perovskite materials are greatly influenced by morphological randomness, making it challenging to precisely control device performance. Future work could explore the combination of template-confined growth patterning techniques and in-situ crystallization control methods to optimize the alignment density and orientation of the NW. 2D perovskites (such as (PEA)_2_PbI_4_), with their quantum well structures and interlayer organic spacers, exhibit high exciton binding energy and enhanced environmental stability. A flexible photodetector fabricated by spray-coating CsPbBr_3_ perovskite nanosheets onto a MXene electrode maintains 85% of its initial photocurrent after 1500 cycles^117^. However, the carrier transport between layers in 2D materials is limited, resulting in slower response times. Future work could focus on designing intercalated molecules to reduce the barriers, combined with in situ photolithography techniques to achieve sub-micron patterning. 3D perovskites, with their high carrier mobility and excellent light absorption capabilities, are the mainstream materials for constructing perovskite PD. The PD based on patterned MAPbBr_3_ perovskite single crystals exhibit a D* as high as 6 × 10^13^ Jones, with an *EQE* of 3900%^120^. However, the environmental sensitivity of 3D materials limits their commercialization. In the future, combining patterning techniques with encapsulation technologies could enhance the long-term stability of the devices. Patterned perovskite materials with different dimensionalities exhibit unique advantages in the field of optoelectronic detection: 0D perovskites offer high stability, 1D perovskites show polarization sensitivity, 2D perovskites exhibit quantum confinement effects, and 3D perovskites provide efficient light absorption. By combining cross-dimensional designs and patterning technologies to address current challenges, their practical implementation in imaging, sensing, and wearable devices can be advanced.

**Table 1:** Summary of the key parameters of perovskite-based photodetectors

Perovskite dimension	Perovskite material	Responsivity (*R*, A W^−1^)	Detectivity (D*, Jones)	Response time (*τ*_r_/*τ*_d_)
0D	hybrid graphene-CsPbBr_3-x_I_x_ NCs^141^	8.2 × 10^8^	2.40 × 10^16^	0.81 s/3.65 s
	CsPb(Br/I)_3_/iGr^142^	20	-	2 s/6 s
	CsPbBr_3_/Au NCs^143^	0.00471	1.68 × 10^9^	0.2 ms/1.2 ms
	Cs_2_AgBiBr_6_^144^	0.0038	1.10 × 10^9^	1.2 ms/2 ms
1D	MAPbI_3_^114^	0.005	-	-
	MAPbI_3_^116^	0.1	1.02 × 10^12^	0.3 ms/0.4 ms
	MAPbI_3_^84^	1.2	2.39 × 10^12^	-
	MAPbI_3_^148^	1.3	2.50 × 10^12^	0.2 ms/0.3 ms
	MAPbI_3_^149^	13.57	5.25 × 10^12^	80 μs/240 μs
	CsPbBr_3_^150^	4400	-	252 μs/300 μs
	MASnI_3_^151^	0.47	8.80 × 10^10^	1.5 s/0.4 s
	FAPbI_3_^65^	0.3	1.10 × 10^9^	19.2 ms/23.9 ms
2D	MAPbI_3_^123^	22	-	20 ms/40 ms
	MAPbI_3_^46^	7	-	~500 μs
	CsPbBr_3_^117^	0.044	6.40 × 10^8^	48 ms/18 ms
	FAPbI_3_^155^	4.8	4.20 × 10^12^	13.7 µs/14.9 µs
	NH_3_(CH_2_)_4_NH_3_PbBr_4_^156^	2.24	1.00 × 10^13^	1.67 ms/1.57 ms
3D	MAPbI_3_^119^	3.49	-	~0.2 s
	MAPbI_3-x_Cl_x_^124^	-	4.00 × 10^14^	180 ns/160 ns
	MAPbI_3-x_Cl_x_^115^	2.17	9.40 × 10^11^	0.48 s/0.26 s
	MAPbBr_3_^120^	16	6.00 × 10^13^	43 µs/36 µs

## Wearable and biomimetic devices based on patterned perovskites

Currently, flexible wearable devices and electrochemical eyes hold great research potential. With advancements in technology, an increasing number of new materials are being applied in these fields. Flexible wearable devices not only enhance the health monitoring experience but also enable more personalized functions. Meanwhile, the emergence of bionic devices, such as electrochemical eyes based on flexible materials and components, opens new directions for the restoration and enhancement of visual functions. The innovative application of patterned perovskite in flexible wearable devices and electrochemical eye components is leading the transformation of the next generation of optoelectronic technologies. Perovskite, due to its excellent optoelectronic conversion efficiency, tunable light absorption properties, and potential for low-cost processing, have become ideal candidates for flexible electronics. The patterning process enhances the local light field and improves charge separation efficiency by constructing regular micro-nano structures on the perovskite thin film. In addition, by incorporating patterning design into the perovskite layer, it helps to evenly distribute stress and reduce the risk of micro-cracks during bending or stretching, ensuring stable output of the device under dynamic deformation. This technology has been preliminarily validated in fields such as flexible solar cells and PD, providing technical support for future integration into smart wearable devices and electrochemical eyes. This technological breakthrough not only promotes the intersection of flexible electronics and biomedical engineering but also lays the scientific foundation for achieving low-power, highly integrated smart wearable devices and bionic vision restoration technologies. It holds strategic significance in reshaping human-machine interaction and medical assistance methods.

### Flexible wearable devices

In recent years, perovskite materials have demonstrated great potential in the field of optoelectronic devices due to their excellent properties, such as high photovoltaic conversion efficiency, tunable band gaps, and low-cost processing. As the demand for flexible electronics and wearable devices continues to grow, flexible perovskite devices are increasingly gaining attention. Specifically, the use of patterning techniques to manipulate the microstructure of perovskite materials allows for enhanced light absorption, improved charge carrier transport, and better mechanical flexibility and environmental stability, making them an ideal solution for high-performance optoelectronic conversion and energy harvesting in flexible wearable devices.

**Fig. 22:**
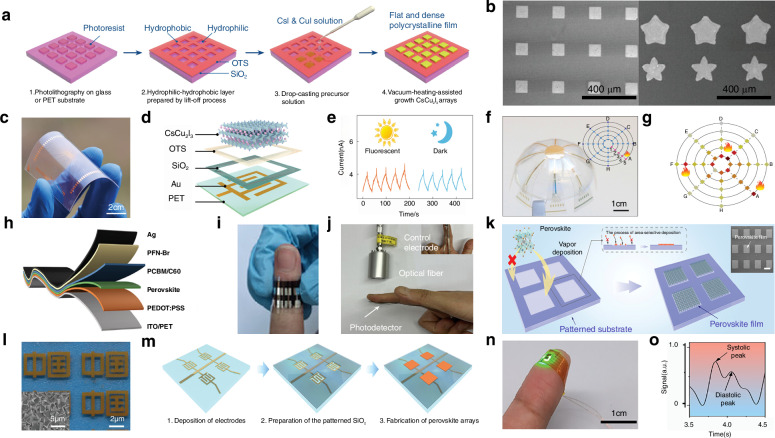
Flexible wearable devices based on patterned perovskite. **a** Schematic of the fabrication procedure for the controlled growth the CsCu_2_I_3_ film. Reproduced with permission^64^, Copyright 2023, Wiley‐VCH GmbH. **b** SEM images of different patterns of CsCu_2_I_3_ films synthesized by the VHADP process. Reproduced with permission^64^, Copyright 2023, Wiley‐VCH GmbH. **c** Optical image of the 10 × 10 flexible solar-blind PDs array. Reproduced with permission^64^, Copyright 2023, Wiley‐VCH GmbH. **d** Schematic of the structure of the solar-blind PD. Reproduced with permission^64^, Copyright 2023, Wiley‐VCH GmbH. **e** Photoswitching properties to the flame under a fluorescent lamp illumination (1.195 mW cm^−2^) and dark environment, respectively. Reproduced with permission^64^, Copyright 2023, Wiley‐VCH GmbH. **f** Digital image of curved solar-blind PD array attached on the hemisphere support. Inset is the corresponding 2D plane diagram. Reproduced with permission^64^, Copyright 2023, Wiley‐VCH GmbH. **g** Current distribution of the curved solar-blind PD array under multi-flame irradiation. Reproduced with permission^64^, Copyright 2023, Wiley‐VCH GmbH. **h** Structure of flexible PD based on tin halide perovskite. Reproduced with permission^164^, Copyright 2024, Wiley‐VCH GmbH. **i** Photo of photoplethysmography test. Reproduced with permission^164^, Copyright 2024, Wiley‐VCH GmbH. **j** Schematic of photoplethysmography test. Reproduced with permission^164^, Copyright 2024, Wiley‐VCH GmbH. **k** Schematic of vapor deposition of perovskite films array. The inset is SEM image of the fabricated perovskite films array. The scale bar is 100 µm. Reproduced with permission^63^, Copyright 2024, The Authors. Advanced Science published by Wiley-VCH GmbH. **l** Photograph of the perovskite patterns of Chinese characters. The inset is the SEM image of the perovskite film. Reproduced with permission^63^, Copyright 2024, The Authors. Advanced Science published by Wiley-VCH GmbH. **m** Schematic of the fabrication process of the perovskite PD arrays. Reproduced with permission^63^, Copyright 2024, The Authors. Advanced Science published by Wiley-VCH GmbH. **n** Photo of the PPG sensor in working conditions. Reproduced with permission^63^, Copyright 2024, The Authors. Advanced Science published by Wiley-VCH GmbH. **o** Amplified single pulse signal diagram. Reproduced with permission^63^, Copyright 2024, The Authors. Advanced Science published by Wiley-VCH GmbH

Using a template-confined growth patterning method, Lu et al. fabricated large-scale patterned CsCu_2_I_3_ perovskite films (Fig. 22a)^64^. The films fabricated on a flexible PET substrate using this approach can achieve a resolution of up to 100 µm. As shown in Fig. 22b, SEM results reveal that the films exhibit a uniform morphology and clear edges. They constructed a 10 × 10-pixel array of flexible solar-blind PDs (Fig. 22c, d). The array achieved a responsivity of 62 A W^−1^ and a minimum detectable power of 6.1 nW cm^−2^, enabling the device to detect extremely weak ultraviolet light. The PD array also exhibited exceptional stability, with almost no performance degradation after continuous operation under ultraviolet light for 8 h. More importantly, the device demonstrated excellent bending stability. After 500 bending cycles at a 30° bend for 20 individual pixels, both the dark current and photocurrent were well maintained (Fig. 22e). The author also developed a flame detection and alarm system consisting of a curved PD array, a signal transmission unit, and a router (Fig. 22f). The curved PD array is designed using a simple origami method, consisting of 40 pixels evenly distributed across 8 arms, and bent into a hemispherical configuration with a diameter of 6 cm. As shown in Fig. 22g, three flames, placed at different spatial directions, are simultaneously positioned around the curved PD array. By mapping the photocurrent, the system is able to identify flames located at different positions. This system is designed for flame detection and location, offering significant contributions to flame condition monitoring and firefighting strategies. With its low power consumption and high sensitivity, it is well-suited for portable and wearable devices, demonstrating the considerable potential of patterned perovskite in solar-blind UV light detection. It is applicable to a wide range of practical applications, including space communication and fire detection systems.

Liu et al. reported a method for fabricating patterned FASnI_3_ tin halide perovskites using template-confined growth patterning technique, incorporating cyanethlamine iodide (CNI) as an additive to enhance the material’s performance^164^. The CNI molecules bind with the Sn^2+^ in the perovskite film, effectively suppressing the oxidation of Sn^2+^. As a result, the proportion of Sn^2+^ in the perovskite film increased from 73% to 93%. This suppression reduces defects in the film, and the average grain size of the perovskite reaches 325 nm, thereby enhancing the optoelectronic performance of the material. They constructed a PD based on patterned FASnI_3_-CNI films, which exhibited exceptional performance, with a dark current as low as 1.04 × 10^-9 ^A cm^−2^, a detectivity of 2.2 × 10^13^ Jones, and a response speed as fast as 2.62 μs. The authors constructed a 32 × 32-pixel PD array, which was capable of detecting light intensities as low as 170 nW cm^−2^. Liu et al. also constructed a flexible photodetector based on tin halide perovskite (Fig. 22h), with a structure of Polyethylene naphthalate 2,6-naphthalene dicarboxylic acid (PEN)/ITO/PEDOT:PSS/FASnI_3_-CNI perovskite/PCBM/C_60_/PFN-Br/Ag. The flexible device exhibited a peak PD *R* of 0.37 A W^−1^ under 785 nm light illumination. Additionally, the device demonstrated a D* exceeding 10^12^ Jones across the 385–905 nm range, with a maximum value of 9.12 × 10^12^ Jones at 785 nm. They applied the flexible PD to photoplethysmogram (PPG) testing to measure human heart rate without extracting any body fluids. As shown in Fig. 22i, j, due to its high detectivity and fast response speed, the heart rate results from the flexible device were comparable to those from devices based on rigid substrates. This demonstrated the successful use of a flexible, non-toxic tin-halide perovskite PD for real-time human heart rate monitoring. Compared to other PD, the FASnI_3_-CNI patterned perovskite PD offers advantages such as lightweight, flexibility, low-temperature fabrication, environmental friendliness, and cost-effectiveness, making it ideal for PPG testing. The heart rate results can be obtained under low-light conditions and zero power consumption, highlighting the great potential of lead-free tin-halide perovskites in wearable human health monitoring.

In addition to the widely used template-confined growth patterning method, vapor deposition patterning has also gained significant attention in the field of flexible sensor fabrication research. Xu et al. reported a method for fabricating patterned CsPbBr_3_ lead halide perovskite using vapor deposition patterning (Fig. 22k)^63^. As shown in Fig. 22l, by adjusting the surface energy of the substrate, the nucleation and growth of the perovskite thin film can be controlled, allowing for the formation of specific patterns in different regions. Using vapor deposition patterning, a perovskite thin film with Chinese character patterns, ~2 µm in thickness, was deposited. Additionally, a high-density perovskite thin film array with a resolution of up to 423 dpi was constructed (Fig. 22m). Xu et al. constructed a photodetector array based on patterned CsPbBr_3_ lead halide perovskite. The device demonstrated excellent optoelectronic performance, with a responsivity of 47.5 A W^−1^, a detectivity of 6.24 × 10^13^ Jones, and a high on/off ratio of 13,887 when a 3 V bias was applied under 450 nm light. The response times were 0.81 ms and 2.03 ms, respectively. Owing to the excellent crystal quality of the perovskite film array produced by the vapor deposition patterning process, the detector demonstrated remarkable long-term stability, maintaining its performance for 12 h under high-humidity conditions. The CsPbBr_3_ perovskite PD array fabricated by the CVD method and a CsPbBr_3_ film array prepared by the solution method as a comparison were both exposed to a harsh environment with an average humidity of 68.6%. After 48 h, the devices made by the solution method showed a significant decrease in photocurrent. In contrast, the CVD-based device maintained stable photocurrents, while the solution-based devices experienced a 25% decrease in photocurrent during the first 12 h. As shown in Fig. 22n, the author designed a pulse monitoring system consisting of a PI film substrate, Au-patterned electrodes, a green LED as the light source, a perovskite film array as the photosensitive layer, and a thin Parylene-C film as the encapsulation layer. This wearable pulse monitoring system, placed on the index finger, utilizes PPG technology to track the pulse by analyzing the light flux. Figure 22o shows the measured PPG signal under an illumination intensity of 8.16 mW cm^−2^. The systolic and diastolic peaks can be observed in each pulse. The heart rate can be monitored under green light illumination with an intensity of 8.16 mW cm^−2^, both at rest and after intense exercise, with values calculated to be 78 and 114 beats per minute, respectively. More importantly, the PPG signal can also be recorded under an illumination intensity as low as 0.055 mW cm^−2^. Owing to the remarkable stability of the perovskite PD, the pulse monitoring system can be worn during daily activities. This example highlights the vast potential of perovskite materials in wearable optoelectronic devices, particularly in health monitoring applications.

Patterned perovskite materials have played a key role in the research of flexible optoelectronic detectors due to their unique crystal growth control and band engineering advantages. Through the precise patterning of perovskite single crystals or nanostructures, device pixelation can be achieved, leading to higher integration density. This process also aids in optimizing crystal orientation, reducing defect density, improving charge carrier mobility, and minimizing dark current, all of which contribute to a significant enhancement in light response speed and detection sensitivity. In recent years, this method has been validated in high-performance optoelectronic integration platforms, with the fabricated arrayed devices exhibiting high responsivity, low noise, and excellent environmental stability, demonstrating broad application prospects in flexible, wearable, and integrated optoelectronic devices.

### Electrochemical eyes

In recent years, perovskite materials have rapidly developed in the fields of optoelectronic detection and more, due to their high photoconversion efficiency, tunable bandgap, low cost, and ease of solution processing. They have also quickly advanced into the field of biomimetic integrated imaging devices. The electrochemical eye is a device that mimics the visual information acquisition and processing mechanism of biological eyes. With the continuous advancement of artificial vision systems and intelligent sensing technologies, electrochemical eyes are gradually becoming a research hotspot for new electronic sensors. In this context, patterned perovskite materials, with their controllable micro-nano structures and exceptional optical response properties, provide a novel technological solution for the development of electrochemical eyes.

**Fig. 23:**
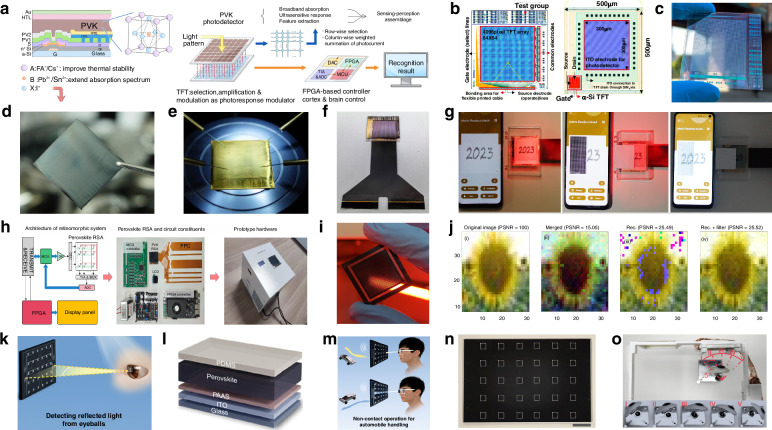
**Electrochemical eyes based on patterned perovskite. a** Schematic and working principle of the retinomorphic computing system based on a 4096-pixel one transistor–one photodetector perovskite retinomorphic sensor array, microcontroller unit, and field-programmable gate array. Reproduced with permission^165^, Copyright 2025, The American Association for the Advancement of Science. **b** Circuit layouts of a 64 × 64 a-Si thin-film transistor (TFT) panel. Reproduced with permission^165^, Copyright 2025, The American Association for the Advancement of Science. **c** Photograph of a 64 × 64 a-Si TFT panel. Reproduced with permission^165^, Copyright 2025, The American Association for the Advancement of Science. **d** Image of a PEDOT:PSS/FA_0.8_Cs_0.2_Pb_0.5_Sn_0.5_I_3_-coated TFT panel. Reproduced with permission^165^, Copyright 2025, The American Association for the Advancement of Science. **e** Image of a full-structure one transistor–one photodetector perovskite retinomorphic sensor array. Reproduced with permission^165^, Copyright 2025, The American Association for the Advancement of Science. **f** Image of a flexible printed cable bonded sample. Reproduced with permission^165^, Copyright 2025, The American Association for the Advancement of Science. **g** Image sensing of handwritten digits 2023 under VIS-NIR monochromic illuminations before and after being partially sheltered by a piece of print paper, respectively. Reproduced with permission^165^, Copyright 2025, The American Association for the Advancement of Science. **h** Architecture and photographs of the complete prototype of perovskite retinomorphic sensor array -based retinomorphic hardware system. Reproduced with permission^165^, Copyright 2025, The American Association for the Advancement of Science. **i** A photo of a perovskite narrowband PD array. Reproduced with permission^166^, Copyright 2023, The American Association for the Advancement of Science. **j** (i) Original image (ii–iv) restored images by different reconstruction methods: (ii) Direct channel merging. (iii) Neuromorphic processing without filtering scheme (iv). Neuromorphic processing with filtering scheme. Reproduced with permission^166^, Copyright 2023, The American Association for the Advancement of Science. **k** Recognition principle of the perovskite-based smart eyeglasses. Reproduced with permission^167^, Copyright 2025, Wiley-VCH GmbH. **l** Schematic of the polyacrylic acid sodium(PAAS) interface layer-assisted perovskite photodetector. Reproduced with permission^167^, Copyright 2025, Wiley-VCH GmbH. **m** Non-contact operation for handling automobiles based on smart eyeglasses. Reproduced with permission^167^, Copyright 2025, Wiley-VCH GmbH. **n** Photograph of the PAAS-MAPbI_3_ device array, containing 5 × 6 detectors. Scale bar: 8 cm. Reproduced with permission^167^, Copyright 2025, Wiley-VCH GmbH. **o** Image of smart glasses equipped with PAAS-MAPbI_3_ devices undergoing a detectable angle of 5°, with the below corresponding angle positions. Reproduced with permission^167^, Copyright 2025, Wiley-VCH GmbH

In early 2025, He et al. reported a retinal biomimetic image sensor based on patterned perovskite, designed to provide intelligent vision functions for exoskeleton robots (Fig. 23a)^165^. They fabricated patterned FA_0.8_Cs_0.2_Pb_0.5_Sn_0.5_I_3_ lead-halide perovskite using a template-confined growth patterning method and constructed a PD. The device exhibited a wide spectral response from 400 nm to 1000 nm, with a single device’s *EQE* reaching 90%, *R* of 0.56 A W^−1^, and D* of 9.11 × 10^12^ Jones. He et al. also constructed a retinal morphology sensor array with a total area of 500 × 500-μm and a resolution of 4096 pixels (Fig. 23b–f). In a low-light environment with an illumination intensity of only 10 μW cm^−2^, the sensor achieved a contrast enhancement of ~620%, demonstrating its excellent performance under weak light conditions (Fig. 23g, h). At the same time, the patterning process also prevents potential voltage crosstalk between adjacent devices. This study demonstrates the application potential of perovskite materials in retinal biomimetic hardware, providing a new solution for intelligent vision in autonomous systems such as exoskeleton robots.

In addition to the wide-spectrum recognition mode, another popular research direction is the construction of red, green, and blue (R/G/B) integrated sensor arrays based on narrow-band patterned perovskites for recognition applications. Hou et al. reported a method for fabricating 2D patterned MAPbX_3_ (X = Cl, Br, I) lead halide perovskite films using template-confined growth patterning technique^166^. They observed a significant imbalance in the electron and hole transport properties of the patterned halide perovskite films. By embedding this perovskite material into structures with different polarities (p-i-n or n-i-p), they achieved narrow-band responses to R/G/B light, enabling the differentiation of different color wavelengths. The device exhibited *R* of 4, 3.5, and 2 mA W^−1^ for red, green, and blue light inputs (700, 550, and 470 nm) under a 0 V bias, while the D* reached 6 × 10^10^ Jones. Based on this, they designed a six-terminal R/G/B vertically stacked patterned perovskite PD array, which can directly capture full-color images without the need for traditional color filter arrays (Fig. 23i). The array features a 32 × 32-pixel layout, with each pixel containing three independent R/G/B channels, totaling 1024 pixels, enabling high-fidelity full-color imaging. The sensor array demonstrates excellent performance in narrow-band response and color differentiation capabilities. The vertically stacked structure, designed based on the patterning process, avoids the complexity of mosaic algorithms in traditional imaging systems, significantly improving imaging efficiency (Fig. 23j). This work provides a systematic innovative approach to materials, devices, and algorithms for novel full-color imaging technology.

Research on electrochemical eyes extends beyond human applications and can also be applied to studies in human-computer interaction. Hu et al. reported a method for fabricating patterned MAPbI_3_ perovskite films using template-confined growth technique, and on this basis, they constructed a perovskite PD array, enabling a demonstration of human-computer interaction based on eye movement control (Fig. 23k, i)^167^. They fabricated patterned perovskite films on glass substrates with pre-deposited ITO electrodes. The patterned sodium polyacrylate interface layer efficiently passivated the defects in the films, ensuring the high quality of the perovskite crystals and their superior optoelectronic performance. Based on this patterned perovskite, the team developed a smart glasses system integrated with perovskite optoelectronic detectors (Fig. 23n). The system demonstrated a switching ratio close to 300, with a *R* reaching 22.09 A W^−1^. Building on this, Hu et al. employed a convolutional neural network algorithm to achieve non-contact, high-precision eye movement recognition with a 5° angular resolution and 99.86% accuracy. They demonstrated the control of a model car’s intricate movements through eye tracking (Fig. 23m, o). This study demonstrates the tremendous potential of patterned perovskite materials in the construction of low-cost, high-performance devices, providing novel and convenient solutions for applications in human-machine interaction, augmented reality, and individual health monitoring.

Patterned perovskite materials are not only suitable for single energy harvesting or sensing functions, but their tunable microstructural properties also facilitate integration with other flexible electronic components to create multifunctional systems, such as bionic vision, environmental sensing, and self-powered devices. In certain cases, researchers have combined patterned perovskite films with flexible circuits and sensors to develop highly integrated flexible optoelectronic sensors and bionic retinal systems. These devices not only show remarkable enhancements in light response speed and sensitivity but also perform exceptionally well in terms of flexibility, durability, and low power consumption, offering a solid technological foundation for the advancement of next-generation wearable smart devices.

## Summary and outlook

We summarized the five most widely used patterning techniques for perovskite materials and their recent advances, and have also considered the latest achievements in the field of PD research based on the dimensional aspects of patterned perovskite films. Perovskite materials are extensively used in the fabrication of PDs because of their outstanding optoelectronic properties and highly tunable band gaps. The variety of patterning techniques also increases their potential for the development of large-scale PD array devices and applications.

In recent years, new methods such as template-confined growth patterning, inkjet printing, vapor deposition growth patterning, and seed-induced growth patterning have rapidly advanced, leading to improvements in the finesse and environmental stability of the patterned perovskite films produced. Novel techniques in traditional photolithography, such as laser-induced modification patterning, have also been developed, progressively improving the precision of patterning while reducing the damage to the materials during processing. The continuous advances in of high-resolution patterning has enabled the fabrication of more refined patterned perovskite films, significantly increasing the miniaturization and integration ability for patterned perovskite PDs. This progress has produced improvements in detector performance and addressed various issues related to optical and electrical crosstalk in integrated devices, thereby advancing the application of perovskite film materials in fields such as photodetection and integrated imaging devices.

The precision of the patterning process directly determines the quality of the photosensitive materials. High-quality patterned perovskite films are widely used in the construction of 0D, 1D, 2D, and 3D PDs. We now systematically summarize in Table 2 the most commonly used methods in perovskite patterning processes, highlighting the advantages and disadvantages of each method, as well as the dimensions of the PDs constructed.

**Table 2:** Summary of the advantages and disadvantages of patterning techniques for constructing perovskite film photodetectors of different dimensions

Patterning technology		Process accuracy	Advantages	Disadvantages	Industrial manufacturing
Template-confined growth patterning	Template-separation assisted patterning	Several hundred nanometers to several micrometers	Simple principle, no damage to the resulting perovskite, and reusable templates	Requires a template, potential damage to the template during demolding, and high costs of precision templates	Widely used in PD, applied to research frontiers (flexible wearable devices, electrochemical eyes), low cost and energy-efficient, high potential for scalability, suitable for constructing flexible devices
	Structural template assisted patterning				
Inkjet printing patterning	Inkjet printing patterning method based on perovskite precursor inks	several micrometers	No template required, high material utilization, capability to fabricate complex patterns, and wide application range	Presence of “coffee ring” effect, difficulty in achieving fine details for complex patterns, and the requirement for sophisticated equipment	High flexibility, minimal material waste, high patterning precision, moderate cost, low energy consumption, high potential for scalability, suitable for constructing flexible devices, but ink composition needs optimization
	Inkjet printing patterning method based on perovskite quantum dot solutions				
Vapor deposition growth patterning	-	several micrometers	No damage to the fabricated patterned perovskite, and the process is relatively simple	The required equipment is expensive, and the patterning accuracy needs improvement	Dominates large-area fabrication of graphene and carbon nanotubes, suitable for organic material coatings, high cost, high energy consumption, suitable for constructing flexible devices
Seed-induced growth patterning	-	Several tens of nanometers	Applicable for single crystal growth	Significant substrate limitations and low patterning accuracy	Emerging technology, high patterning accuracy, good substrate compatibility, moderate cost, low energy consumption, suitable for constructing flexible devices
Conventional photolithogra-phy patterning	Focused ion beam lithography patterning	Several hundred nanometers to several micrometers	No need for templates, widest application range, high precision, programmable operation, and mature process	Causes damage to perovskite materials and involves high equipment costs	Extremely high patterning accuracy, very high cost, high energy consumption, can cause damage to the thin film surface, suitable for constructing flexible devices
	Electron beam lithography patterning				
	Laser direct writing patterning				
	Laser-induced modification patterning				

Several dimensional PDs constructed from patterned perovskite films have now achieved exceptional detection performance. These studies underscore the significant importance and broad prospects of patterned perovskite materials in the development of high-performance PDs. They also highlight the crucial value of advances in patterning technology for improving device performance and integration. Despite the remarkable achievements in perovskite patterning technology and the construction of photonic devices based on patterned perovskite films, there are still challenges to be overcome in device fabrication and future commercial applications.

First, the perovskite materials currently used are mostly lead-containing, and the toxicity of the Pb significantly limits the practical applications of their patterned PDs. Although research on lead-free perovskite is increasingly popular and fabrication techniques for their patterning are gradually being developed, the optoelectronic performance of their PDs still does not match that of PDs based on lead halide perovskites. Further research is therefore essential. Using alternative elements, such as Cu, to replace Pb is a viable solution. Additionally, actively developing techniques to recycle Pb from lead halide perovskite materials could reduce the harmful effects of Pb on living organisms and the environment.

Second, the resolution of the patterned perovskite film plays a decisive role in the performance of the final optoelectronic detector. Currently, most perovskite patterning methods cannot simultaneously achieve high resolution and fabrication efficiency. For example, the patterned perovskite produced by template-confined growth can achieve high resolution, but the quality of the fabricated devices is heavily influenced by the template design. Exploring the incorporation of amphiphilic polymers or ZnO nanorods to stabilize the template structure, combined with PDMS replication technology to create inverted honeycomb-like micro-nano composite structures, could enhance the pattern complexity. Inkjet printing patterning has significant advantages in the fabrication of complex patterns, but the “coffee ring” effect generated during the process limits its application. The resolution can be improved by optimizing the ink formulation (such as increasing viscosity or using multi-solvent systems) to meet the requirements for high-precision device fabrication. Additionally, combining electrodynamics techniques to precisely control droplet ejection with an electric field to enhance pattern quality is a promising research direction with great potential. The vapor deposition patterning method is suitable for large-area uniform film formation, but its patterning accuracy needs to be improved. Recently, solvent-induced vapor polymerization technology has enabled patterning on flexible substrates such as cellulose and hydrogel without the need for oxidants, offering a new approach for wearable electronics. Photolithography patterning suffers from issues such as performance degradation of perovskite due to residual photoresist and solvent etching. The use of sacrificial layers combined with selective solvent removal can effectively reduce the damage to perovskite caused by direct exposure to the photolithography environment. Furthermore, the protective layer formed after photolithography can block water and oxygen corrosion, enhancing the long-term stability of the device and enabling self-optimized encapsulation. In the foreseeable future, the exploration of fabrication processes and techniques for high-resolution, high-quality patterned perovskite will remain a popular research direction in the development of patterned perovskite PDs.

Third, while significant progress has been made in the research of perovskite PD, studies on large-scale patterned perovskite photodetector arrays are still in the early stages. There is still considerable room for development in the integration and size of the constructed arrays. The standardization and cost reduction of large-scale array manufacturing are crucial for the practical application of patterned perovskite PDs. Although patterned perovskite photodetector arrays have already developed exciting practical applications, such as artificial retinas, many current low-cost patterning methods, such as inkjet printing, still face the issue of low resolution. Improvements in the drying process (such as controlling Marangoni flow) can reduce film defects and are compatible with roll-to-roll production, promoting low-cost, large-scale manufacturing. The template-confined growth patterning method faces issues such as insufficient mechanical stability of the template, high template costs, and limited reusability. Exploring self-assembly coatings to reduce template adhesion, combined with template methods and laser annealing to minimize subsequent etching steps, would help advance the application of template-based methods in industrial manufacturing. The photolithography method, which offers high patterning resolution, heavily relies on the light source, and the energy cost of the light source is high, making it difficult for large-scale production. Introducing PMMA or SiO_2_ spacer layers to isolate the photoresist from the perovskite, combined with LDW technology, can enable submicron patterning in a non-vacuum environment, reducing costs and effectively advancing the application of photolithography in the industrial production of patterned perovskite optoelectronic devices. The challenge of balancing large-scale array resolution with production costs significantly impacts the practical application of optoelectronic devices in everyday life.

Finally, long-term stability is a crucial factor determining whether patterned perovskite materials can be practically applied. Perovskite materials are prone to decomposition when exposed to environments containing moisture and oxygen. Once decomposition occurs, the performance and lifespan of patterned perovskite devices will significantly deteriorate. Some patterning techniques can create a protective barrier on the surface of perovskite films, serving a similar function to encapsulation. This barrier not only isolates moisture and oxygen molecules from the external environment but also allows for the regulation of thermal conductivity and stress distribution by optimizing the geometric shape of the pattern, thereby reducing the risk of crystal fracture or phase transition caused by localized thermal expansion. In addition, the most widely used technique to address the decomposition issue of perovskites is encapsulation. Currently, perovskite encapsulation can be broadly categorized into three different types: encapsulation based on oxide core-shell nanostructures, encapsulation based on polymers, and encapsulation based on metal-organic frameworks. By integrating patterning and encapsulation processes to form protective barriers on the surface of perovskite materials, the degradation rate of perovskites in humid conditions can be significantly slowed, thereby enhancing the device’s longevity.

Overall, advances in patterning technology are crucial for the development of high-performance patterned perovskite PDs, because high-quality patterned perovskite serves as the foundation for these devices. To date, PDs constructed from patterned perovskite materials have been explored in research fields such as electrochemical eyes and medical imaging devices, yielding promising results. However, problems such as the presence of toxic elements and long-term device stability remain to be addressed. We anticipate that future perovskite thin-film patterning technologies will become increasingly advanced, and this with the development of perovskite photonic devices will propel the application of perovskite materials in optoelectronics and microelectronics. Ultimately, this will lead to the practical implementation of perovskite-based PDs and integrated devices in real products and everyday life, ushering in a new phase of development for perovskite optoelectronic devices.

## Data Availability

The data that support the findings in this study are available from the corresponding author upon reasonable request.
